# The Ribosomal DNA Loci of the Ancient Monocot *Pistia stratiotes* L. (Araceae) Contain Different Variants of the 35S and 5S Ribosomal RNA Gene Units

**DOI:** 10.3389/fpls.2022.819750

**Published:** 2022-03-03

**Authors:** Anton Stepanenko, Guimin Chen, Phuong T. N. Hoang, Jörg Fuchs, Ingo Schubert, Nikolai Borisjuk

**Affiliations:** ^1^Jiangsu Key Laboratory for Eco-Agricultural Biotechnology Around Hongze Lake and Jiangsu Collaborative Innovation Centre of Regional Modern Agriculture and Environmental Protection, School of Life Sciences, Huaiyin Normal University, Huai’an, China; ^2^Leibniz Institute of Plant Genetics and Crop Plant Research (IPK), Gatersleben, Germany; ^3^Faculty of Biology, Dalat University, Đà Lạt, Vietnam

**Keywords:** *Pistia stratiotes*, FISH, gene organization, molecular evolution, 35S rDNA, 5S rDNA

## Abstract

The freshwater plant water lettuce (*Pistia stratiotes* L.) grows in warm climatic zones and is used for phytoremediation and biomass production. *P. stratiotes* belongs to the Araceae, an ecologically and structurally diverse early monocot family, but the phylogenetic relationships among Araceae members are poorly understood. Ribosomal DNAs (rDNAs), including the 35S and 5S rDNA, encode the RNA components of ribosomes and are widely used in phylogenetic and evolutionary studies of various plant taxa. Here, we comprehensively characterized the chromosomal locations and molecular organization of 35S and 5S rDNA genes in water lettuce using karyological and molecular methods. Fluorescence *in situ* hybridization revealed a single location for the 35S and 5S rDNA loci, each on a different pair of the species’ 28 chromosomes. Molecular cloning and nucleotide sequencing of 35S rDNA of *P. stratiotes*, the first representative Araceae *sensu stricto* in which such a study was performed, displayed typical structural characteristics. The full-length repeat showed high sequence conservation of the regions producing the 18S, 5.8S, and 25S rRNAs and divergence of the internal transcribed spacers ITS1 and ITS2 as well as the large intergenic spacer (IGS). Alignments of the deduced sequence of 18S rDNA with the sequences available for other Araceae and representatives of other clades were used for phylogenetic analysis. Examination of 11 IGS sequences revealed significant intra-genomic length variability due to variation in subrepeat number, with four types of units detected within the 35S rDNA locus of the *P. stratiotes* genome (estimated size 407 Mb/1C). Similarly, the 5S rDNA locus harbors gene units comprising a conserved 119-bp sequence encoding 5S rRNA and two types of non-transcribed spacer (NTS) sequences. Type I was classified into four subtypes, which apparently originated via progressive loss of subrepeats within the duplicated NTS region containing the 3’ part of the 5S rRNA gene. The minor Type II NTS is shorter than Type I and differs in nucleotide composition. Some DNA clones containing two or three consecutive 5S rDNA repeats harbored 5S rDNA genes with different types of NTSs, confirming the mosaic composition of the 5S rDNA locus.

## Introduction

Water lettuce (*Pistia stratiotes*) belongs to a monospecific genus in the subfamily Aroideae of the ecologically and structurally diverse ancient monocot family Araceae *sensu lato* (also known as aroids), a group of 118 genera comprising approximately 3,800 species (Henriquez et al., 2014). Most aroids are tropical and subtropical species, but some members inhabit temperate regions, displaying broad habitat diversity, including geophytes, epiphytes, helophytes, climbers, and floating aquatics (Croat, 1988; Mayo et al., 1998; Keating, 2004). Bayesian analysis of divergence times based on multiple fossil and geological calibration points revealed that the *Pistia* lineage is 76–90 million years old (Renner and Zhang, 2004).

Water lettuce, which floats in fresh water, displays rapid, mostly vegetative propagation, and high biomass accumulation. In many locations, these features qualify *P. stratiotes* as an invasive species that is difficult to eliminate (Paolacci et al., 2018). However, *P. stratiotes* plants have tremendous potential for water bioremediation due to their capacity for fast and efficient assimilation of nitrogen and phosphate, heavy metals, and other water contaminants. Therefore, *P. stratiotes*, like the aquatic duckweeds (Acosta et al., 2021), has been used to remediate different types of wastewater (Zimmels et al., 2006; Rezania et al., 2016; Zhou and Borisjuk, 2019). For example, Lu et al. (2010) determined that water lettuce was superior to most other plants for efficient wastewater bioremediation due to its capability to annually remove 190–329 kg/ha of nitrogen and 25–34 kg/ha of phosphorus. Additionally, its high amounts of proteins and carbohydrates make *P. stratiotes* a valuable biomass resource for use as a green fertilizer or soil amendment (Kodituwakku and Yatawara, 2020) or as feedstock for the production of nitrogen-doped biochar (Zhang et al., 2021).

Ribosomal DNA (rDNA) plays a pivotal role in organisms by producing the RNA components required to form ribosomes (Moss and Stefanovsky, 2002; Appels et al., 2021; Hemleben et al., 2021). In plants, as in most eukaryotes, the rDNA encodes four ribosomal RNAs (rRNAs), which serve as the major structural and functional components of the ribosome. Plant rRNA genes typically occur in two types of loci: 35S rDNA loci containing three tightly linked rRNA genes (18S-5.8S-25S), which are transcribed by RNA Polymerase I into a 35S rRNA precursor; and 5S rDNA loci encoding 5S rRNA transcribed by RNA Polymerase III. The 35S and 5S rDNA loci have clusters of tandemly repeated units composed of conserved coding sequences and diverse intergenic spacers (IGSs) (Volkov et al., 2003). Due to its high copy number, its conserved coding sequence, and its more rapidly evolving spacer sequences, rDNA has become a favorite subject of studies related to plant systematics, evolution, and biodiversity and is used as a genome-specific marker in allopolyploids and hybrids (Borisjuk et al., 1988; Stadler et al., 1995; Mahelka et al., 2017).

To date, the DNA sequence data for the Araceae family have mostly been obtained from chloroplasts and mitochondria (Renner et al., 2004; Rothwell et al., 2004; Cusimano et al., 2011; Henriquez et al., 2014; Choi et al., 2017; Gao et al., 2018; Tian et al., 2018). With the exception of the whole-genome sequences of five species of the remotely related duckweeds (Acosta et al., 2021), little information is available about the nuclear genes of Araceae species, including their rDNA. The nuclear genomes of several Araceae species have been studied by examining the 35S and 5S rDNA loci using fluorescence *in situ* hybridization (FISH) (Sousa et al., 2014; Lakshmanan et al., 2015; Sousa and Renner, 2015; Vasconcelos et al., 2018), and a few nuclear rDNA sequences, represented by a single 25S rDNA sequence for *Spathiphyllum wallisii* (Zanis et al., 2003) and several sequences for 18S rDNA and ITS1-5.8S-ITS2, have been deposited in GenBank.

In this study, to gain a deeper understanding of the molecular organization and functionality of plant rDNAs and of the phylogenetic relationships and evolutionary history of the Araceae, we sequenced and examined the chromosomal localizations of *Pistia stratiotes* rDNAs. The obtained data include the entire nucleotide sequence of 11 35S rDNA repeat units (18S-ITS1-5.8S-ITS2-25S rDNA and the IGS) and sequences of 63 clones representing the variability of the 5S rDNA units in the *P. stratiotes* genome.

## Materials and Methods

### Plant Material

A *Pistia stratiotes* plant, designated as isolate TB-1, was purchased from an online seller (taobao.cn) and cultivated in fresh water under laboratory conditions for molecular and cytological analysis. The species’ identity was confirmed by DNA barcoding using primers specific for chloroplast intergenic spacers atpF-atpH (ATP) and psbK-psbL (PSB) as previously described (Borisjuk et al., 2015).

#### Genome Size Measurement

For flow cytometric genome size measurements roughly 0.5 cm^2^ of fresh leaf tissue of *P. stratiotes* and *Raphanus sativus* cv. Voran (2C = 1.11 pg; Genebank Gatersleben, accession number: RA 34) as internal reference standard were co-chopped with a sharp razorblade in a Petri dish using the “CyStain PI Absolute P” reagent kit (Sysmex-Partec) according to manufacturers’ instructions. The samples were filtered through a 50 μm mesh and measured on a CyFlow Space flow cytometer (Sysmex-Partec). The DNA content (pg/2C) was calculated based on the values of the G1 peak means and the corresponding genome size (Mbp/1C), according to Dolezel et al. (2003).

#### Mitotic Chromosome Preparation

The plants were grown in nutrient solution (Appenroth et al., 1996) until daughter plants with new roots had developed. The root tips were collected and treated in 2 mM 8-hydroxylquinoline at 37°C for 2 h and then fixed in fresh 3:1 (absolute ethanol: acetic acid) for 48 h. The fixed samples were washed twice in 10 mM Na-citrate buffer pH 4.6 for 10 min each before and after softening in 2 mL PC enzyme mixture (1% pectinase and 1% cellulase in sodium-citrate buffer) for 120 min at 37°C, prior to maceration and squashing in 60% acetic acid. After freezing in liquid nitrogen, the slides were treated with pepsin, (50 μg pepsin/mL in 0.01 N HCl, 5 min at 37°C), post-fixed in 4% formaldehyde in 2x SSC (300 mM Na-citrate, 30 mM NaCl, pH 7.0) for 10 min, rinsed twice in 2x SSC, 5 min each, dehydrated in an ethanol series (70, 90, and 96%, 2 min each) and air-dried.

#### Ribosomal DNA Probe Labeling

The *A. thaliana* BAC clone T15P10 (Arabidopsis Biological Resource Center, United States), labeled by nick-translation was used as 35S rDNA probe. Genomic DNA of the giant duckweed (*Spirodela polyrhiza*) was used for PCR-amplification of 5S rDNA with a primer pair listed in Hoang et al. (2019) and designed according to the 5S rDNA sequence of *Glycine max* (Gottlob-McHugh et al., 1990). The PCR product was used as template for PCR-labeling to generate the 5S rDNA FISH probe.

The 5S rDNA probe was labeled with Cy3-dUTP (GE Healthcare Life Science), and the 35S rDNA probe with Texas Red-12-dUTP (Life Technologies) and precipitated as described (Hoang and Schubert, 2017).

#### Fluorescence *in situ* Hybridization

Probes were denatured at 95°C for 5 min and chilled on ice for 10 min before adding 10 μL of each probe per slide. Then, the mitotic chromosome preparations were denatured together with the probes on a heating plate at 80°C for 3 min, followed by incubation in a moist chamber at 37°C for at least 16 h. Post-hybridization washing and signal detection were done as described (Lysak et al., 2006) with minor modifications. Widefield fluorescence microscopy for signal detection followed Cao et al. (2016). The images were pseudo-colored and merged using Adobe Photoshop software ver.12 (Adobe Systems).

#### Cloning and Sequence Analysis of 35S Ribosomal DNA

For analysis of rDNA genes, total DNA was isolated from the fresh biomass of *Pistia stratiotes* using the CTAB method (Murray and Thompson, 1980) modified according to Borisjuk et al. (2015). To clone the 35S rDNA, genomic DNA of *P. stratiotes* was digested with *Xba*I + *Mfe*1 restriction enzymes (Takara, China) and fractionated by agarose gel electrophoresis. DNA fragments of 2–6 kb were purified from the gel using AxyPrep^^TM^ DNA Gel Extraction Kit (Axygen, United States) and ligated into pUC18 plasmid, digested with *Xba*I + *EcoR*1. Following transformation of the plasmids into *E. coli*, about 230 of the obtained colonies were screened by PCR using three sets of primers (Supplementary Figure 1). One set of primers, specific for internal part of 18S rRNA gene was used to select clones containing the 18S-5.8S-25S rDNA; two additional pairs of primers, one specific for the 3′-end of 25S rRNA gene (Clo25Sfor and Clo25Srev), and one specific for the 5′-end of 18S rRNA gene (Clo18Sfor and Clo18Srev) were used for selecting clones containing the end of 25S rDNA, the intergenic spacer (IGS) and the 5′part of the 18S rDNA. The colony PCR screening resulted in selecting two clones containing the coding rDNA portion, Pi-rDNA-1 and Pi-rDNA-2, and one clone with the IGS, Pi-IGS-1. The isolated plasmids were custom sequenced by Sangon Biotech (Shanghai, China) using the combination of standard forward and reverse pUC18 primers and a number of the insert internal primers designed according the progress of the sequencing (Supplementary Table 1).

Based on the obtained sequence of Pi-IGS-1, primers specific for 18S and 25S rRNA genes were used to amplify the IGS region. The generated DNA fragments, were cloned into the vector pMD19 (Takara, Dalian, China) and the fragments of selected clones were checked by digestion using restriction enzymes *Eco*RI + *Hin*dIII and *Eco*RI + *Pst*I (Takara, Dalian, China). Ten clones with rDNA fragments of different length were custom sequenced (Sangon Biotech, Shanghai, China) using a combination of standard forward and reverse sequencing primers and a range of internal primers designed according the original sequence of the Pi-IGS-1 (Supplementary Table 1). The obtained nucleotide sequences were analyzed using the CLC Main Workbench (Version 6.9.2, Qiagen) software. The resulted sequences of the *P. stratiotes* rDNA fragments are deposited in the GenBank.

#### Cloning and Sequence Characterization of 5S Ribosomal RNA Genes

For analysis of *P. stratiotes* 5S rRNA genes, the specific DNA fragments were amplified from genomic DNA by PCR using two pairs of primers specific for 5S rRNA gene sequence DW-5S-F/DW-5S-R and cn-5S-for/cn-5S-rev (Supplementary Table 1) as previously described (Chen et al., 2021). In order to increase the chance of amplifying DNA fragments with multiple 5S rDNA units, the elongation time of PCR was prolonged to 1 min 30 s. The generated DNA fragments, cloned into the vector pMD19 (Takara, Dalian, China) were custom sequenced (Sangon Biotech, Shanghai, China), and the obtained nucleotide sequences were analyzed using the CLC Main Workbench (Version 6.9.2, Qiagen) software. Upon sequence analysis of the obtained clones, a second round of PCR amplification using primers specific for the revealed NTS sequences was performed. The primers used for amplification of 5S rDNA units are listed in Supplementary Table 1.

#### *In silico* Analysis of the Ribosomal DNA Sequences

The obtained *P. stratiotes* sequences for 18S rRNA were used to examine the phylogenetic relationships primarily within the Araceae family, but also with the representatives of other plant clades. The corresponding sequences available for Araceae and representative species for monocots, magnoliids and eudicots were extracted from the GenBank^1^ by blasting with the *P. stratiotes* sequences as a query, with cut off *E*-value equal to 0.05. The maximum-likelihood phylogenetic trees were constructed using NGPhylogeny webservice accessible through the https://ngphylogeny.fr using MAFFT Multiple Sequence Alignment and FastME algorithm (Lefort et al., 2015; Lemoine et al., 2019). iTOL^2^ was used for displaying and annotating the generated phylogenetic trees (Letunic and Bork, 2021).

For detection of the DNA regions likely to fold into G-quadruplex structures, we have primarily used the *pqsfinder* prediction tool (Labudová et al., 2020) available at the website,^3^ with further verification by the G4Hunter algorithm (Brázda et al., 2019), freely available at DNA Analyzer server.^4^

## Results

### Characterization of the Chromosome Set and Visualization of 35S and 5S Ribosomal DNA Loci in *Pistia stratiotes*

**FIGURE 1:**
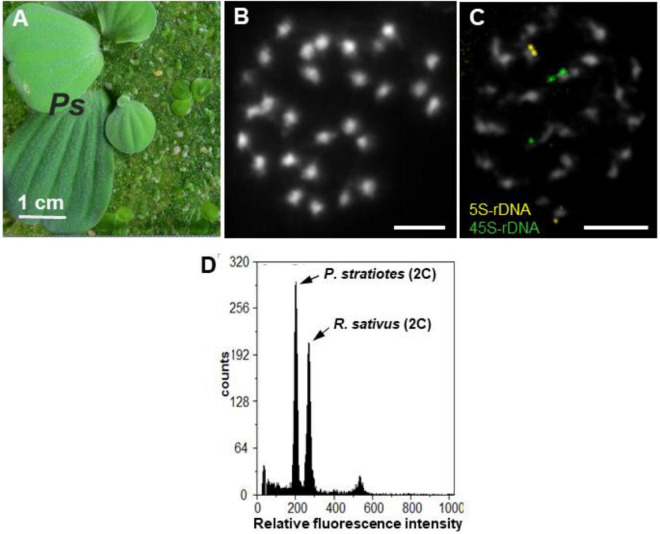
*P. statiotes*, its chromosomes and nuclear DNA content. **(A)** Whole *P. stratiotes* plant (***Ps***) in the presence of different duckweed species. **(B)** Complete meristematic metaphase, with 28 small DAPI-stained chromosomes (scale bar: 5 μm). **(C)** FISH signals for 5S (yellow) and 35S rDNA (green) loci at one end of one pair of chromosomes each (scale bar: 5 μm). **(D)** Histogram of nuclear DNA of *P. stratiotes*, and *Raphanus sativus* as an internal reference standard.

The identity of the *Pistis stratiotes* TB-1 isolate used in this study (Figure 1A) was confirmed by examining the chloroplast DNA barcodes ATP (intergenic spacers atpF-atpH, Ac# OL435916) and PSB (intergenic spacers psbK-psbL, Ac# OL435917). Alignment of the obtained sequences showed that these sequences shared 100 and 99.6% similarity, respectively, with the corresponding ATP and PSB spacers of the *P. stratiotes* chloroplast genome deposited in GenBank (accession no. NC_048522).

The root tip meristems of *P. stratiotes* displayed a number of small chromosomes (2n = 28) after DAPI staining (Figure 1B). Due to their small size, structural details, such as primary (centromere) or secondary [nucleolus-organizing region (NOR)] constrictions, of these chromosomes were barely recognizable. Therefore, whether the chromosomes of this species are mono- or holocentric remains unknown. FISH with 5S and 35S rDNA probes uncovered one pair of chromosomes, each with terminal signals (Figure 1C), supporting the diploid nature of the species. Flow cytometric measurements of isolated nuclei (Figure 1D) revealed a nuclear genome size of 407 Mbp/1C (unreplicated haploid chromosome complement).

### Nucleotide Sequence Analysis of 35S Ribosomal DNA

#### DNA Sequences Encoding 18S-5.8S-25S Ribosomal RNA

Analysis of the sequences of various duckweeds species, the only group of related aquatic plants with relatively well-characterized 18S and 25S rDNA genes (Tippery et al., 2015), and our restriction mapping of 35S rDNA in *S. polyrhiza* (Michael et al., 2017) revealed unique conservative restriction sites for *Xba*I in the 18S rDNA and for Mfe1 in the 25S rDNA. Our strategy to clone the entire 35S rDNA repeat unit was built on this finding, assuming a similar situation for *Pistia.* We characterized the entire 35S rDNA repeat sequence by sequencing three cloned genomic Mfe1 + *Xba*I restriction fragments (Supplementary Figure 1). Sequence comparison of the two clones, Pi-rDNA-1 and Pi-rDNA-2 (Supplementary Figure 2), containing part of the 35S rDNA repeat encoding 18S-5.8S-25S ribosomal RNA, revealed high nucleotide conservation with just eight single nucleotide polymorphisms (SNPs) over a length of 5,365 bp. Six of the detected SNPs were T↔C transitions, one was a C↔G transversion, and one was a nucleotide deletion located in ITS1, 5.8S, and 25S rRNA coding genes, with no variations in the sequences for 18S rDNA or ITS2. Due to this low sequence divergence, we used the 18S and 25S rDNA sequences of the clone Pi-rDNA-1, supplemented with the missing parts of the 5′-end of the 18S rDNA and the 3′-end of the 25S rDNA from clone Pi-IGS (Supplementary Figure 1), resulting in a 5,868-bp-long sequence covering the 18S-ITS1-5.8S-ITS2-25S rDNA for further analysis.

BLAST analysis showed that the *P. stratiotes* 18S rDNA sequence has the highest similarity (99.5%) to a previously sequenced but not published *Pistia* gene (accession no. AF168869), 96.98% similarity to 18S rDNA of the Araceae species *Orontium aquaticum* (Qiu et al., 2000), 97.5% to *Calla palustris* (accession no. AF168829), 96.99% to *Spathiphyllum wallisii* (accession no. AF207023), 96.14% to *Gymnostachys anceps* (accession no. AF069200), 97% to *Symplocarpus nipponicus* (accession no. MT247907) (Do et al., 2020), and 95.31–96.58% to the 18S rDNA sequences of the 36 duckweed species (Tippery et al., 2015; Hoang et al., 2020), whose 18S rDNA sequences are available in GenBank. The 18S rDNA-based phylogenetic tree is shown in Supplementary Figure 3. The *P. stratiotes* 5.8S rDNA sequence showed many more BLAST hits in the Araceae family than the 18S rDNA. The hit with the highest score (98.16%) was for the *Amorphophallus elliottii* gene (accession no. KR534451), followed by *Lasia spinosa* (Yeng et al., 2016) and numerous duckweed species (Tippery et al., 2015). When the query included ITS1 and/or ITS2 in addition to the 5.8S rDNA sequence, the list of meaningful BLAST hits primarily included species representing the *Schismatoglottidoideae* clade (genera *Aridarum*, *Bakoa*, *Hottarum*, *Ooia*, and *Piptospatha*) and the genus *Amorphophallus.* According to the advanced phylogenetics based on chloroplast and mitochondria DNA data (Henriquez et al., 2014), the *Schismatoglottidoideae* and *Amorphophallus* together with *Pistia* belong to the Aroidae subfamily of the Araceae. BLAST analysis of the 25S rDNA sequence of *P. stratiotes* primarily revealed the homologous sequences of duckweeds deposited by Tippery et al. (2015) and the 25S rDNA sequence of *Spathiphyllum wallisii* (Zanis et al., 2003), demonstrating the scarce representation of Araceae in the GenBank database.

#### Sequence Organization of the 35S Ribosomal DNA Intergenic Spacer Region of *Pistia stratiotes*

**FIGURE 2:**
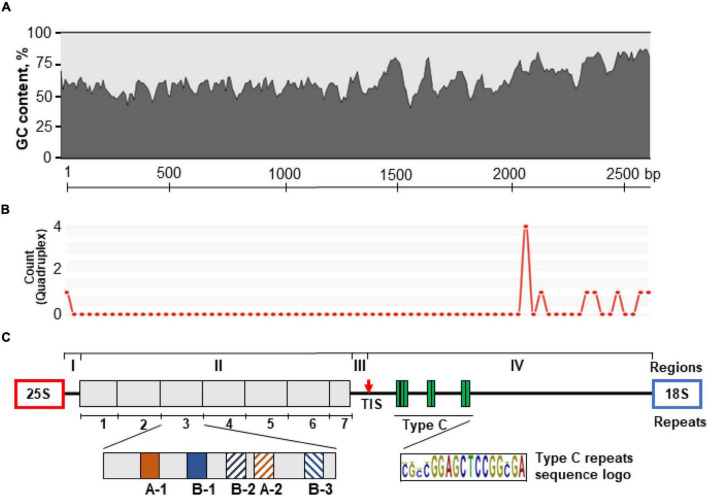
Schematic representation of the molecular architecture of the 35S rDNA IGS of *P. stratiotes* obtained by sequencing the genomic clone Pi-IGS (accession no. OL409040). **(A)** Pattern of G + C nucleotide distribution along the IGS sequence calculated using window size of 45 nucleotides. **(B)** Patterns of G-quadruplex structures predicted for the IGS sequence using window size of 50 nucleotides; the heights of the peaks indicate the relative strength of each G-quadruplex structure. **(C)** The IGS is divided into four regions (I–IV); region II contains seven repeats (1–7) composed of subrepeats A-1, A-2, B-1, B-2, and B-3; region III has a transcription initiation site (TIS), marked by a red arrow; region IV, which corresponds to the 5′-ETS, contains Type C subrepeats with the consensus sequence CGCCGGAGCTCCGGCGA.

Sequencing of the Pi-IGS clone (Supplementary Figures 1, 4) revealed an IGS region of 2,644 bp with many features of molecular architecture previously described for other plants (Appels and Dvořák, 1982; Delcasso-Tremousaygue et al., 1988; Borisjuk and Hemleben, 1993; Borisjuk et al., 1997), as well as the classic plant rRNA transcription initiation site (TIS) signature TATAGGGGG located in the middle of the IGS, 1,270 bp upstream of the 18S rRNA gene. The *P. stratiotes* IGS has a relatively high average overall GC content of 61.7%, with an approximately equal percentage of AT and GC within the first half of the sequence and an irregular GC pattern in the second part of the IGS, reaching more than 75% close to the beginning of the 18S rRNA gene (Figure 2A). This GC-enriched part of the IGS is also predicted to form G-quadruplex structures, which might be involved in regulating the transcription and/or processing/stability of the transcribed 35S rRNA precursor (Figure 2B).

Sequence analysis showed that the *P. stratiotes* IGS can be subdivided into four distinct structural regions (I–IV, Figure 2C). Region I, with a length of 51 bp, represents a unique sequence, with two pyrimidine-rich motifs, CCCTGTCCCACCACCC and CCCCACTCACCCC, starting at nucleotide positions 1 and 39 relative to the end of the 25S rDNA gene (Supplementary Figure 4). Such motifs are believed to serve as transcription termination sites.

Region II (1,218 bp, GC content 56.3%) consists of seven units of repeated sequences. Each repeat is composed of subrepeats organized in a specific pattern of two major subrepeat types (A and B). This pattern resembles that found in rice, with the 253–264-bp subrepeats composed of three types of short related DNA elements (Cordesse et al., 1993). The Type A and B *Pistia* subrepeats are normally 19 bp long, with a certain level of divergence, which roughly divides them into subtypes A-1, A-2, B-1, B-2, and B-3. Each repeat contains two Type A subrepeats and three Type B subrepeats arranged in a A-1/B-1/B-2/A-2/B-3 pattern, with some sequence erosion in repeat 1, while repeat 7 is represented only by a combination of A-1 and B-1, as revealed by nucleotide alignment of the repeat sequences (Figure 2C). There is also a certain degree of variation between sequences of the same subtype, especially those located close to the repeat zone borders (Supplementary Figure 5). Repeat 1 (168 bp) is shorter than repeats 3–6 (191 bp) and demonstrates a higher degree of sequence variation. The Type A subtypes of repeat 1 are incomplete and are 13 and 16 nucleotides in size. The 5′ end of repeat 2 (191 bp) is also quite polymorphic relative to other repeats. The last repeated unit, repeat 7, is incomplete, is 91 bp long, and contains only two subrepeats, which is characteristic of the first parts of repeats 1–6.

**FIGURE 3:**
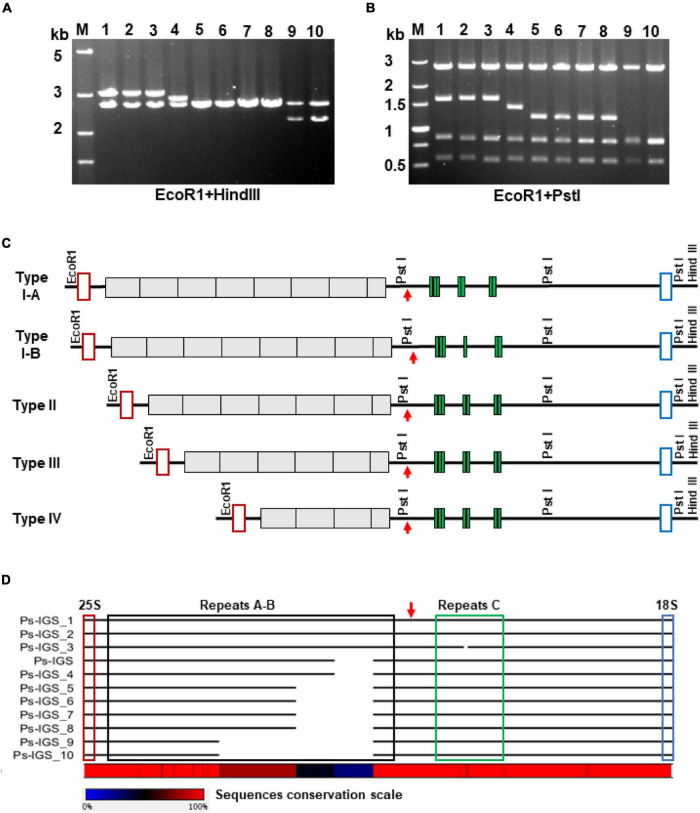
Polymorphism of the *P. stratiotes* 35S rDNA repeat units, as revealed by analysis of IGS sequences in 10 clones containing PCR-amplified fragments. **(A,B)** Length variation of the cloned IGSs, as revealed by analyzing restriction fragment polymorphisms produced by digesting plasmid DNA with the restriction enzymes *Eco*RI plus *Hin*dIII **(A)** and *Eco*RI plus *Pst*I **(B)** and visualized by agarose gel electrophoresis. M—DNA fragments of molecular weight marker DL5000 (Takara) with their length in kilobases on the left site of the gel; numbers 1–10 above the gel images correspond to DNA clones Ps-IGS_1 to Ps-IGS_10. **(C)** Molecular structures of rDNA fragments depicted in A and B, based on full-length nucleotide sequences of the clones. The 10 clones were divided into four major groups based on the sequencing results: Type I–IV, depending on number of repeats in A-B and C. Clones Ps-IGS_1 and Ps-IGS_2 represent Type IA; Ps-IGS_3 represents Type IB; Ps-IGS_4 represents Type II; Ps-IGS_5, Ps-IGS_6, Ps-IGS_7, and Ps-IGS_8 represent Type III; Ps-IGS_9 and Ps-IGS_10 represent Type IV. Open red rectangles mark 25S rDNA sequences; gray blocks mark A-B repeats, green blocks mark C-repeats; open blue rectangles mark 18S rDNA sequences; the TIS is marked by a red arrow. **(D)** Simplified representation of the sequence alignment of the clones; the full nucleotide alignment and the sequences’ accession numbers are shown in Supplementary Figure 4.

Region III, which is 202 bp long (GC content 61.4%), does not have any subrepeats and harbors a TIS with a signature typical for the majority of plant species examined (King et al., 1993; Volkov et al., 2003; Krawczyk et al., 2017). Region IV (1,155 bp) corresponds to the transcribed 5′-ETS and is characterized by the presence of three clusters of GC-rich C subrepeats, separated by unique sequences of lower GC percentage (Figure 3). The first cluster contains three subrepeats, and the other two contain only two subrepeats. The region of C-repeats is followed by a unique sequence characterized by higher GC content compared to the other regions.

To gain insight into the possible intragenomic heterogeneity of the individual repeats of 35S rDNA in *P. stratiotes*, we amplified the entire IGS region by PCR using primers specific for the 25S and 18S genes, followed by cloning and characterization of 10 individual clones by restriction enzyme analysis and nucleotide sequencing. Digestion of the clones with restriction enzymes *Eco*RI and *Hin*dIII, which cut out the entire insert, yielded fragments between 2 and 3 kb (Figure 3A). Additional digestion with *Eco*RI and *Pst*I, the latter having two recognition sites within the IGS according to the sequence of the original Ps-IGS clone (Supplementary Figure 4), revealed two invariable fragments covering the IGS region between the TIS and 18S rRNA gene and a fragment of variable size corresponding to the region of A-B subrepeats upstream of the TIS according to the scheme in Figure 2. Sequencing of the 10 clones revealed further details about their molecular architecture. Each clone starts with 53 nucleotides of the 25S rDNA and ends with 41 nucleotides of the 18S rDNA, with four major variants of IGS between the rRNA coding sequences. The three longest fragment variants in clones Pi-IGS_1, Pi-IGS_2, and Pi-IGS_3, which represent *P. stratiotes* IGS Type I, characterized by eight repeats upstream of the TIS, could be subdivided into subtypes I-A and I-B based on the copy numbers of C-repeats downstream of the TIS (Figure 3).

The two IGSs classified into Type I-A, Pi-IGS_1 and Pi-IGS_2, are 2,837 and 2,833 bp long, respectively, and contain seven copies of C-repeats. By contrast, the IGS of clone Pi-IGS_3, which is 2,816 bp long, is classified as Type I-B, with six copies of C-repeats. The Type II IGS, represented by clone Pi-IGS_4, with seven repeats upstream of the TIS and a length of 2,643 bp, is very similar to the IGS of genomic clone Pi-IGS (2,644 bp long; Figure 3); the two sequences differ by just 11 SNPs, 7 of which are T↔C transitions. The Type III IGS is represented by four almost identical sequences featuring six repeats upstream of the TIS, with a length of 2,453 (clone Pi-IGS_5) and 2,452 bp (clones Pi-IGS_6, Pi-IGS_7, and Pi-IGS_8). Altogether, the four sequences share 41 SNPs dominated by 17 A↔G and 16 T↔C transitions.

The shortest IGS, Type IV, is represented by two clones 2,069 and 2,067 bp long (clones Pi-IGS_9 and Pi-IGS_10) with four repeats upstream of the TIS. Alignment of the obtained IGS sequences of *P. stratiotes* 35S rDNA revealed very high sequence conservation within each of the four IGS types as well as between types, with the major variations related to the number of internal repeats upstream of the TIS (Figure 3D and Supplementary Figure 4).

### Characterization of the 5S Ribosomal DNA

To characterize the molecular structure of the 5S rDNA, we cloned PCR products amplified with two pairs of primers designed to cover neighboring 5S rDNA genes with the NTS between them and sequenced individual clones. The analysis of chimeric sequences reconstructed from parts of neighboring genes might lead to inaccurate conclusions (Galián et al., 2014a), so we used only the through-sequenced 5S rDNAs in subsequent analyses. Based on the sequencing results, we designed an additional pair of primers specific for the NTS sequences and used them to confirm the specific arrangements of the 5S rDNA units (Supplementary Figure 7). In total, we sequenced and analyzed 50 clones containing complete 5S rDNA repeats, composed of a sequence encoding 5S rRNA and an adjacent NTS, including 18 clones containing 2 5S rDNA units and 5 clones containing 3 sequential 5S rDNA units.

#### 5S Ribosomal RNA Gene Sequences

**FIGURE 4:**
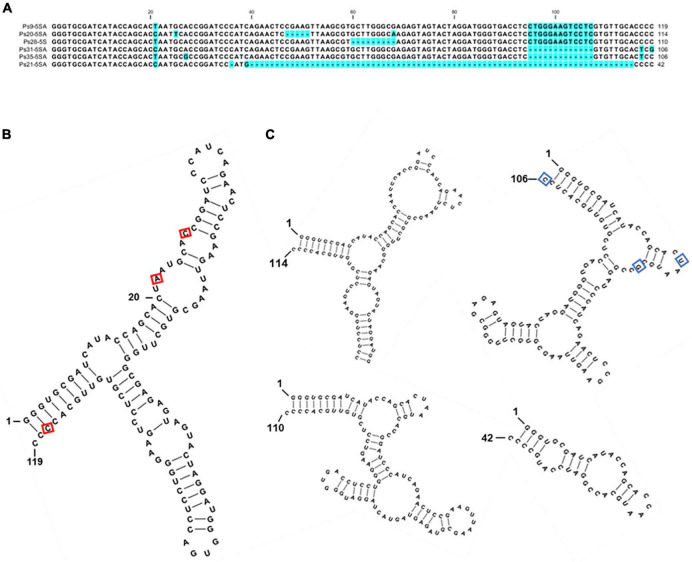
Sequence variants of 5S rDNA identified in the genome of *P. stratiotes.* **(A)** Nucleotide alignment of the *P. stratiotes* 5S rDNA variants. **(B)** Predicted secondary structure of the full-length 119-nucleotide 5S rRNA. The positions of three nucleotide substitutions occurring at high frequencies are marked with a red rectangle. **(C)** Secondary structures of the transcripts derived from pseudogene variants 114, 110, 106, and 42 nucleotides long. The 106 nucleotides long pseudogene variant is represented by sequence Ps35-5SA, with the positions of variable nucleotides in clone Ps31-5SA marked by blue rectangles. The free energy values of the predicted RNA secondary structures are: 119 bp: ΔG = –48.6 kcal/mol; 114 bp: ΔG = –38.9 kcal/mol; 110 bp: ΔG = –38.4 kcal/mol; 106 bp: ΔG = –33.6 kcal/mol; 42 bp: ΔG = –10.4 kcal/mol.

Sequence analysis of the clones revealed 78 gene units containing the full-length sequence of the 5S rDNA gene. Only these through-sequenced 5S rDNA genes were used for further analysis (Supplementary Figure 8). Among these, 73 units contained a 5S rRNA gene sequence 119 bp long, which is characteristic of these sequences in the majority of plants analyzed (Vandenberghe et al., 1984; Zanke et al., 1995; Tynkevich and Volkov, 2014; Simon et al., 2018; Chen et al., 2021). The gene displayed several sequence variations, with 31 random nucleotide substitutions (mostly T↔C and G↔A transitions) and three more regular transitions: at positions 21 (T↔C, with a 85.9% frequency of T), 29 (G↔A, with a 73.1% frequency of A), and 117 (T↔C, with a 53.8% frequency of T). In the predicted 5S rRNA secondary structure, the substitutions at nucleotides 21 and 27 localize to the loops and are thus unlikely to affect rRNA folding, whereas the substitution at position 117 might make the intra-molecular pairing of the ends of the molecule more relaxed (Figure 4). Computer-aided folding of five shorter 5S rRNA gene sequences [114, 110, 106 (two), and 42 bp] revealed secondary structures highly divergent from that obtained for the full-length gene (119 bp), suggesting that these genes are not functional and represent pseudogenes (Figure 4C).

#### Variants of the 5S Ribosomal DNA Non-transcribed Spacers

**FIGURE 5:**
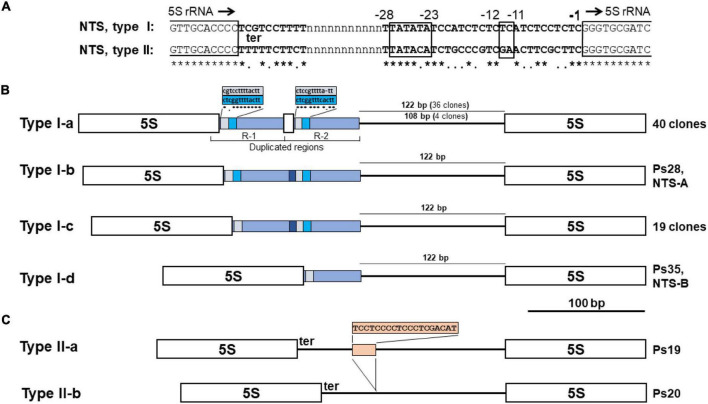
NTS sequence variants of 5S rDNA in the genome of *Pistia stratiotes*. **(A)** Regulatory DNA elements downstream and upstream of the 5S rRNA genes. The end and beginning of 5S rRNA genes are marked by open boxes. Position -1 marks the first nucleotide upstream of the 5S rDNA transcription start; black arrows indicate the direction of gene transcription. **ter**, transcription terminator. The boxed nucleotide position -11 to -12 marks the usual location of the GC dinucleotide; positions -23 to -28 mark the TATA-like motif. **(B)** Schematic representation of 5S rDNA units with Type I NTSs. Open boxes define the sequences of 5S rRNA genes. **(C)** Schematic representation of 5S rDNA units with Type II NTSs. Open boxes define the sequences of 5S rRNA genes.

Among the clones, we identified 64 full-length NTS sequences separating the 5S rDNA genes. These NTS sequences could be separated into two groups based on length and the nucleotide motifs at their 5′-ends (which usually define the termination of transcription of the 5S rRNA) and their 3′-ends (containing DNA elements that modulate gene transcription) (Hemleben and Werts, 1988; Cloix et al., 2003). Members of the major group of 61 NTS sequences (Type I) are longer and contain a TCGT motif at their 5′-terminus, which follows the end of 5S rRNA gene (Figure 5A). This represents a divergence from the classic TTTT transcription termination signal found in the majority of plants; however, a TCGT motif has been documented for some other monocot species: rice (*Oryza sativa*; GenBank: CP054686.1, positions 13743700–13751999) and *Scilla scilloides* (accession no. LC213012) and the eudicot *Gossypium* (Cronn et al., 1996). The minor group (Type II) is represented by two of the 64 NTSs (clones Ps19 and Ps20), which start with the classic 5S rDNA terminator motif TTTTT found in most of the plants analyzed, including the duckweeds *Spirodela polyrhiza* (Borisjuk et al., 2018) and *Landoltia punctata* (Chen et al., 2021). Types I and II also have a slightly different nucleotide arrangement at their 3′-termini in front of the 5S rRNA gene (Figure 5A).

The Type I NTSs can be further classified into four subtypes according to their length and molecular architecture (Figure 5B). The characteristic feature of the major subtype I-a, represented by 40 NTS sequences, is the tandem duplication of a 68-bp region that includes 15 bp of the 3′-terminal sequence of the 5S rRNA gene. The entire R-1 and R-2 sequences differ by a three-nucleotide deletion and five nucleotide substitutions. The 15-bp stretch of the 5S rRNA gene sequence in both duplicated regions (R-1 and R-2 in Figure 5B) is followed by a nearly perfect duplication of 13 nucleotides containing the transcription termination signal (Figure 5). The next part of the type I-a NTS is represented by either of two types of sequences: (i) a 122-nucleotide sequence with 17 nucleotide substitutions between 36 clones, or (ii) a 108-bp sequence with only a single T↔C transition found in four clones (Figure 5 and Supplementary Figure 8).

Subtype I-b, represented by a single sequence of the clone Ps28, differs from subtype I-a sequences in that the 15-bp piece of the 5S rRNA gene sequence in R-2 was replaced by the shorter sequence GCAAGTCCT, with no obvious sequence homology to the 5S rRNA gene. Compared to subtype I-b, subtype I-c (20 sequences) is shorter by 13 bp, lacking one of the two duplicated elements following the 5S rRNA gene sequence. Subtype I-d (represented by a single sequence of Ps35, NTS-B) differs from subtype I-c due to the elimination of all sequence elements of the R-2 derivative.

The Type II NTS with the classic TTTT transcription termination site is represented in our survey by two clones of a size similar to that of Type I-d (Figure 5C). Both clones have almost identical sequences (Supplementary Figure 8). The main difference is that a TC-rich insertion of 19 nucleotides provides the longer variant with an additional G4 structure (Supplementary Figure 9).

#### The 5S Ribosomal DNA Unit Types Are Intermingled Within the Locus

**FIGURE 6:**
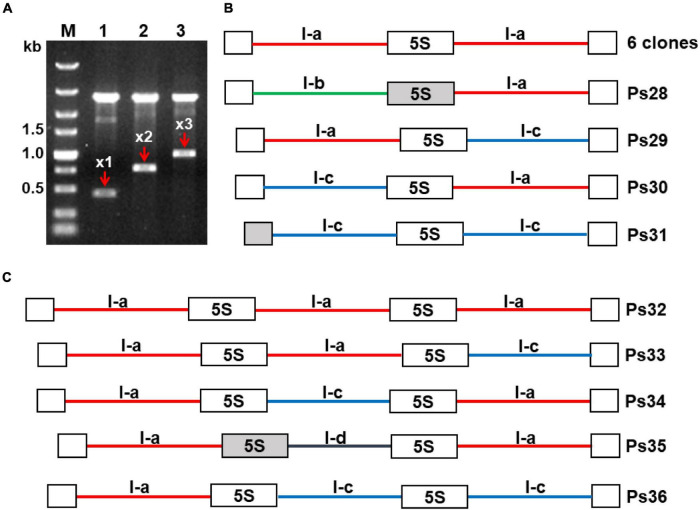
Modes of repeat arrangement revealed in clones containing multiple 5S rDNA units. **(A)** Agarose gel showing digested plasmids containing DNA fragments composed of one (x1), two (x2), and three (x3) units of 5S rDNA in the representative clones Ps20, Ps25, and Ps35, respectively. **(B)** Schematic representation of the arrangement of rDNA units with NTS types I-a, I-b, and I-c in clones containing two 5S rDNA repeats. **(C)** Schematic representation of the arrangement of rDNA units with NTS types I-a, I-c, and I-d in clones containing three 5S rDNA repeats. All depicted DNA clones are in 5′–3′ orientation. Open boxes represent full-length (119 bp) 5S rDNA genes; gray boxes in clones Ps28, Ps31, and Ps35 represent 5S rDNA pseudogenes. The six identical clones with double NTSs of type I-a are represented in GenBank by the sequence Ps22 with accession number OL409056. The accession numbers of other sequences are as follows: Ps28—OL409060; Ps29—OL409057; Ps30—OL409058; Ps31—OL409059; Ps32—OL409061; Ps33—OL409062; Ps34—OL409063; Ps35—OL409064; Ps36—OL409065.

The finding that *P. stratiotes* harbors a single 5S rDNA locus, as revealed by FISH, suggests that gene units with different NTS types are arranged in a certain order within this locus. Indeed, sequence analyses of the 10 clones containing two 5S rDNA repeats and 5 clones with three consecutive repeated units demonstrated different modes of unit arrangement. Of the 10 double unit clones, 7 contained rDNA units of the same type: 6 clones contained the dominant I-a type NTSs, and 1 contained type I-c units. The three remaining clones showed a mixed unit arrangement: I-a/I-c (clone Ps29), I-b/I-a (Ps28), and 1-c/1-a (Ps30) (Figure 6).

Of the five triple clones, one contained three 5S rDNA units with the same I-a type NTS (clone Ps32), and the others displayed different arrangements of NTS types I-a, I-c, and I-d: a/a/c (clone Ps33), a/c/a (clone Ps34), a/d/a (clone Ps35), and a/c/c (clone Ps36) (Figure 6C). To confirm the mixed patterns of type I-a and I-c NTSs, we developed a pair of specific primers for these NTS types and cloned and sequenced the amplified fragments. In total, we obtained six clones containing a 179-bp type I-c NTS, a 119-bp 5S rDNA, and an 81-bp type I-a NTS, confirming that 5S rDNA units with the two most common types of NTS are intermingled within the locus. Of special note is clone Ps21, containing a 5S rDNA pseudogene of 42 bp followed by an atypically short NTS (118 bp) and a regular 119-bp 5S rDNA sequence (Supplementary Figure 10). The sequence of this NTS is more similar to a Type II NTS than a Type I NTS but starts with TCGA motifs.

## Discussion

The identity of the Chinese *Pistia stratiotes* isolate examined in this study (Figure 1A) was confirmed by chloroplast DNA barcodes ATP and PSB. Alignment of the obtained sequences revealed 100 and 99.6% similarity, respectively, to the corresponding ATP and PSB spacers of the chloroplast genome deposited in GenBank (accession no. NC_048522) obtained from an ecotype that originated from North America. The high sequence similarity of the chloroplast DNA barcodes, combined with the 99.5% similarity of 18S rDNA sequences from the Chinese (this study) and US (accession no. AF168869) isolates, suggests a relatively low genetic variability between ecotypes of this mostly vegetatively propagating aquatic plant.

### Genome Size, Chromosome Number, and Localization of Ribosomal DNA Loci

In agreement with previous reports (Blackburn, 1933; Subramanyam, 1962; Krishnappa, 1971; Geber, 1989), our study revealed that *P. stratiotes* has a set of 2n = 28 small chromosomes. The estimated haploid genome size of 407 Mbp, is roughly 39% larger than previously reported by Geber (1989; 249 Mbp) The suggested reason for this discrepancy is that, in contrast to the presented data, previous measurements were performed by Feulgen microdensitometry. In any case, the obtained values are in the range of 100 Mbp—1 Gbp, typical for many small and fast-growing angiosperms (Leitch et al., 2019). FISH revealed a single terminal locus for both 5S and 35S rRNA genes, supporting the diploid status of *P. stratiotes*. Single rDNA loci are common in several diploid angiosperms, with 51.38% of these plants having a single 5S rDNA locus and 35.5% having a single 35S rDNA locus according to Garcia et al. (2017).

Karyologic data were available only for approximately one-quarter of the 3,300–3,600 species belonging to Araceae *sensu lato* (including Lemnaceae as Lemnoidea) (Cusimano et al., 2012), an ancient and very diverse family (Mayo et al., 1997; Boyce and Croat, 2013; Vasconcelos et al., 2018) whose history has been traced back to the early Cretaceous period (Friis et al., 2004), probably overlapping with of dinosaurs. Chromosome numbers ranging from 2n = 10 to 2n = 168 were reported for this group (Cusimano et al., 2012). However, increasing interest in the phylogeny and evolution of monocots in general (Barfod et al., 2010), and the Araceae family in particular (Cusimano et al., 2011) during the current decade, has stimulated research on Araceae cytogenomics, which led to the localization of 5S and 35S rDNA by FISH for several representative species. Two or four 35S rDNA loci have been mapped in six ornamental species of the genera *Anthurium*, *Monstera*, *Philodendron*, *Spathiphyllum*, *Syngonium*, and *Zantedeschia* (Lakshmanan et al., 2015). Mapping of 5S rDNA and 35S rDNA in 10 species of the genus *Typhonium* (Sousa et al., 2014) and in 14 species with chromosome numbers of 2n = 14 to 2n = 60, representing 11 different genera (Sousa and Renner, 2015), resulted in the visualization of 1 pair of 5S rDNA sites in subterminal or interstitial regions and (predominantly) 4 loci of 35S rDNA. By contrast, analysis of 29 species of *Philodendron* and 5 species of *Thaumatophyllum*, with chromosome numbers ranging from 2n = 28 to 2n = 36, revealed one or two loci for 5S rDNA and a wide range of 1–9 chromosomes with FISH signals for 35S rDNA (Vasconcelos et al., 2018). Localization of rDNA in 11 species representing all 5 genera of duckweed, with a 2n chromosome number ranging from 36 to 82, revealed 1–3 loci for 5S rDNA and 1 or 2 loci for 35S rDNA (Hoang et al., 2019). These increasing amounts of data on chromosomal rDNA representation should be extended by further investigating the functionality and molecular evolution of the Araceae 5S and 35S rDNA genes.

### Molecular Organization and Evolution of 35S Ribosomal DNA Repeats in *Pistia stratiotes*

Deciphering the full-length nucleotide sequence of 35S rDNA in *P. stratiotes*, for the first time for the Araceae *sensu stricto*, revealed a molecular architecture typically found in other plant taxa (Hemleben and Zentgraf, 1994), with conserved sequences encoding 18S, 5.8S, and 25S rRNAs and diverse internal transcribed spacers, ITS1 and ITS2, and the IGS separating the coding sequences.

BLAST analysis of the rDNA gene sequences revealed the highest similarities (∼99.5%) to the single entry of 18S rDNA genes from *P. stratiotes* (sampled in the United States) in GenBank, demonstrating high sequence conservation among different *P. stratiotes* ecotypes. The phylogenetic tree based on 18S rDNA sequences (Supplementary Figure 3) groups *P. stratiotes* together with other Araceae species, with a certain distance from duckweeds, showing potential for further resolving phylogenetic relationships within the Araceae family (Tippery et al., 2021). No entry for the 5.8S rDNA of *P. stratiotes* was found in GenBank. The best hits for the 5.8S rDNA gene were the homologous sequences of *Amorphophallus elliottii*, a species that belongs to the same subfamily, *Aroideae*; however, the 5.8S rDNA sequence is too short and conserved for building a reliable phylogenetic tree. By contrast, the ITS1 and ITS2 sequences are more variable, making them a relatively popular tool in plant phylogenetic studies (Alvarez and Wendel, 2003). However, up to date there are not too many Araceae ITS sequences available in the GenBank. For example, when using “megablast” option for highly similar sequences, the *Pistia* ITS queries hit the homologous sequences representing a single genus of the whole Araceae family—*Amorphophallus*.

The IGS of *P. stratiotes* 35S rDNA has a structure typically observed in plants, comprising four structural/functional regions: (i) a 3′-end external transcribed sequence (3′-ETS) with the presumed transcription termination site; (ii) a region of repeats, often functioning to enhance 35S rRNA precursor transcription; (iii) a region containing the transcription initiation site (TIS); and (iv) the 5′-external transcribed spacer (ETS), which stretches to the beginning of the sequence encoding 18S rRNA.

The arrangement of 3′-ETS in the 35S rDNA IGS of *P. stratiotes* (region I, Figure 2C) resembles that described for other monocotyledonous species such as rice, maize, sorghum, and *Brachypodium distachyon* (Krawczyk et al., 2017) as well as dicots (Borisjuk et al., 1997; Volkov et al., 2017). Between region II and the TIS (region III) with the sequence TATTATAGGGG, closely resembling that of other dicot and monocot plants (Volkov et al., 2003; Huang et al., 2017), is a unique 78-bp sequence that does not match any homologs deposited in GenBank. This one more time highlights the fact that no IGS sequences of related Araceae species are available for comparison in GenBank, considering that the IGS sequences upstream of TIS demonstrated significant similarity in other groups of species representing the same family, such as maize, wheat, and rice of Poaceae (Cordesse et al., 1993) or potato, tobacco, and tomato of Solanaceae (Borisjuk et al., 1997). The 5′-ETS (region IV), a 1,255-bp region in *Pistia*, contains clusters of 17-bp repeats downstream of the TIS and a gradually increasing GC content toward the beginning of the 18S rDNA, averaging 75.1% within the 600-bp region adjacent to the 18S rDNA gene. This GC-enriched region may form G4 structures (Figure 2), possibly contributing to the regulation of transcription and replication of rDNA (Havlová and Fajkus, 2020). BLAST analysis of the *Pistia* 5′-ETS sequence upstream of the 18S gene, an IGS region of high similarity between species of the same family (Cordesse et al., 1993; Borisjuk et al., 1997; Volkov et al., 2017), did not reveal any homologs in GenBank. This was surprising, as numerous sequences of this region from duckweed species (Tippery et al., 2015) were deposited in GenBank, and the classification based on morphological observations assumes a close relationship between duckweeds and core aroids including *Pistia* (Maheshwari, 1958). However, the finding agrees with the notion that duckweeds are not very closely related to the core Araceae and should be treated as a separate sister family (Lemnaceae) of Araceae (Tippery and Les, 2020; Acosta et al., 2021) rather than as the subfamily Lemnoidae within the Araceae (French et al., 1995; Cusimano et al., 2011).

Our data on the intragenomic heterogeneity of the 35S rDNA in *P. stratiotes*, which we generated by sequencing one genomic clone and 10 random IGS fragments obtained by PCR, shed additional light on the genome organization and evolution of plant rDNA. Nucleotide alignment of the obtained sequences revealed significant variation in IGS size, primarily due to variation in the number of repeats upstream of the TIS, which is well documented to occur at inter species level (Chang et al., 2010; Huang et al., 2017; Hemleben et al., 2021), between cultivars (Polanco and De La Vega, 1997) or between different rDNA loci of the same genome (Sáez-Vásquez and Delseny, 2019; Appels et al., 2021). Apart from the variation in repeat numbers, the IGSs demonstrate considerable sequence homogeneity, with 95 SNPs among the 11 IGS sequences (∼3 SNPs per kbp) (Supplementary Figure 4).

Remarkably, the variation in repeat copy number exclusively involves the most conserved repeated units in the middle of the track (Figure 3), while the more divergent flanking repeats remained intact in all 11 IGS variants. The area of the most homogenized repeats likely represents a recombination hot spot that is responsible for the IGS length variation in *P. stratiotes*. Another point to highlight is that the single 35S rDNA locus visualized by FISH contains at least five repeat variants (most likely more, considering that the 11 clones characterized here may not cover the entire IGS sequence variability). This observation supports the growing amount of data showing that the 35S rDNA repeats within a locus are not fully homogenized (Galián et al., 2014b; Sims et al., 2021).

### Molecular Architecture of *Pistia stratiotes* 5S Ribosomal DNA

To the best of our knowledge, this work represents the first molecular study of 5S rDNA in Araceae considering the related duckweeds, for which data are available (Borisjuk et al., 2018; Hoang et al., 2020; Chen et al., 2021), as a separate family. The sequencing of multiple 5S rDNA gene units, containing 78 sequences of full length 5S rRNA gene and 64 full-length intergenic spacers of *P. stratiotes*, revealed a molecular arrangement common for the majority of analyzed plants (Zhu et al., 2008), with a conserved 119-bp sequence for 5S rRNA and a variable non-transcribed spacer (NTS). Certain nucleotide variations, mostly T↔C and G↔A transitions, which are often observed in other plants (Cronn et al., 1996), were detected in the 5S rDNA of *P. stratiotes*. Of special interest is the T↔C transition at position + 117 of the coding region. The 37 5S rRNA genes containing T at the + 117 position are followed by NTS Types I-c and 1-d, whereas the genes with C at this position are linked with NTS Types I-a and II.

In addition, analysis of the gene coding region showed some variants with deletions. Based on the deduced rRNA secondary structure (Figure 4), these variants appear to be pseudogenes. Similar degraded 5S rDNA sequences have been described for many eukaryotic organisms, especially fishes (Rebordinos et al., 2013; Barman et al., 2016), crustaceans (Perina et al., 2011), and plants (Sergeeva et al., 2017; Volkov et al., 2017). Other molecular pathways than those leading to “concerted evolution” likely contribute to shaping the landscape of plant 5S rDNA (Nei and Rooney, 2005).

Similar to the 5S rDNA of the duckweeds *S. polyrhiza*, *S. intermedia*, and *L. punctata* (Hoang et al., 2020; Chen et al., 2021), the *P. stratiotes* gene units can be divided into two major classes (Type I and II) and their subtypes (Figure 5) with slightly different nucleotide arrangements at their 5′- and 3′-termini. These differences affect G4 structures (Supplementary Figure 9), and can potentially influence a gene’s functionality (Yadav et al., 2017; Havlová and Fajkus, 2020).

The intragenomic variability of 5S rDNA in *P. stratiotes* is quite intriguing, considering that a single 5S rDNA locus per genome was visualized by FISH (Figure 1). Even more interesting is our finding about the arrangement of different gene units within the locus uncovered by sequencing clones containing double and triple 5S rDNA units (Figure 6) and of PCR clones generated with primers specific for different types of NTSs (Supplementary Figure 11). The data summarized in Figure 6 suggest that different types of 5S rDNA units are randomly interlinked with each other along the rDNA stretch, forming various combinations between neighboring units, according to their frequency in the genome (Supplementary Figure 8). Fortunately, among these multiunit clones, we also identified less frequent types of rDNA units (NTS types 1b and 1d) and even one unit with a defective 5S rDNA sequences. These data further detail the mosaic arrangement of 5S rDNA revealed earlier in rice (Zhu et al., 2008, GenBank ID: CP054686.1, range: 13,743,700–13,751,999 bp), wheat (Sergeeva et al., 2017), and the duckweed *Landoltia punctata* (Chen et al., 2021), providing new insights into 5S rDNA organization and arrangement in plants.

Taken together, due to the generation of multiple sequences of both 35S and 5S rDNA units, our study sheds new light on the intra-genomic variability of rDNA and provides novel findings about the evolution of these genes in plants. In general, the newly obtained data support the recent trend suggesting separate modes of molecular evolution for 35S and 5S rDNA (Mahelka et al., 2013; Volkov et al., 2017).

## Conclusion

Our combined molecular, cytogenetic, and phylogenetic data provide comprehensive characterization of rDNA loci of the ancient monocot plant *P. stratiotes*, representing the Araceae family. Our findings confirm the high conservation of sequences encoding 18S, 5.8S, 25S, and 5S rRNAs.

Nucleotide sequencing of multiple clones containing the most variable parts of rDNA, the IGS of 35S rDNA and the NTS of 5S rDNA, uncovered the scale of intra-genomic and intra-locus variation. Our data support the idea of a mosaic arrangement of multiple variants of 35S and 5S rDNA units in single loci as the rule rather than the exception. The *P. stratiotes* 35S rDNA locus displayed at least four length variants of the gene, which differ in the number of repeats within the IGS, but not in the promoter or transcribed sequences. The 5S rDNA locus displayed at least six types of functional gene units, intermingled with each other and with pseudogenes. Our findings confirm the notion that sequence homogenization through “concerted evolution,” together with crossovers at recombination hot spots in IGS repeats, are major molecular forces shaping the 35S rDNA arrays in plants. At the 5S rDNA locus, an evolutionary shortening of the dominant type of gene units through gradual loss of repeated NTS elements apparently took place and likely generated non-functional pseudogene variants, best described by the “birth-and-death” model. Thus, our findings suggest that 35S rDNA and 5S rDNA in plants evolve via different mechanisms.

## Data Availability Statement

The datasets presented in this study can be found in online repositories. The names of the repository/repositories and accession number(s) can be found in the article/Supplementary Material.

## Author Contributions

IS and NB conceived the study. AS and GC conducted molecular analysis of rDNA and generated all rDNA sequencing data. PH and JF performed chromosome analysis. AS, IS, and NB organized, analyzed the data, and wrote the manuscript. All authors contributed to the article and approved the submitted version.

## Conflict of Interest

The authors declare that the research was conducted in the absence of any commercial or financial relationships that could be construed as a potential conflict of interest.

## Publisher’s Note

All claims expressed in this article are solely those of the authors and do not necessarily represent those of their affiliated organizations, or those of the publisher, the editors and the reviewers. Any product that may be evaluated in this article, or claim that may be made by its manufacturer, is not guaranteed or endorsed by the publisher.

## Footnotes

1
https://www.ncbi.nlm.nih.gov

2
https://itol.embl.de

3
https://pqsfinder.fi.muni.cz/

4
http://bioinformatics.ibp.cz

## Supplementary Material

Supplementary Figure 1 General organization of plant 35S rDNA repeats and representation of the cloned fragments of *Pistia stratiotes* rDNA units characterized in this study. Two genomic DNA regions encoding the major parts of 18S-5.8S-25S rRNA genes (Pi-rDNA-1 and Pi-rDNA-2) and a DNA region containing the 3′-part of the 25S rDNA, intergenic spacer (IGS), and 5′-part of the 18S rDNA (Pi-IGS-1) were cloned as *Xba*I + *Mfe*I restriction fragments. Ten additional fragments covering the IGS region (Pi-IGS_1 to Pi-IGS-10) were produced by PCR using primers specific for the coding sequences of 25S rRNA (F1) and 18S rRNA (R1) and through-sequenced with internal primers F2, F3, R2, and R3.

Supplementary Figure 2 Sequence alignment of *P. stratiotes* rDNA units encoding 18S-5.8S-25S rRNAs represented by genomic clones Pi-rDNA-1 (accession no. OL375700) and Pi-rDNA-2 (accession no. OL375701). Variable residues are highlighted in light blue. Blue rectangles mark 18S rDNA sequences; yellow rectangles mark 5.8S sequences; red rectangles mark 25S rDNA sequences.

Supplementary Figure 3 Phylogeny of monocots, eudicots, and magnoliids derived from 18S rDNA sequences. The phylograms shows a maximum likelihood tree obtained using different available 18S rDNA sequences from different plant clades. Branch lengths represent the expected numbers of substitutions per nucleotide site.

Supplementary Figure 4 Sequence alignment of IGS variants revealed in *P. stratiotes* 35S rDNA. Variable residues and regions are highlighted in light blue. Red rectangle marks 25S rDNA sequences; black rectangles mark A-B repeats; green rectangles mark C repeats; blue rectangle marks sequences of 18S rDNA; the TIS is marked by a red arrow; *Pst*I restriction sites are marked by black arrows. The sequences’ accession numbers are as follows: Ps-IGS: OL409040; Ps-IGS_1: OL409041; Ps-IGS_: OL409042; Ps-IGS_3: OL409043; Ps-IGS_4: OL409044; Ps-IGS_5: OL409045; Ps-IGS_6: OL409046; Ps-IGS_7: OL409047; Ps-IGS_8: OL409048; Ps-IGS_9: OL409049; Ps-IGS_10: OL409050.

Supplementary Figure 5 Sequence alignment of the seven repeats (1–7) comprising IGS zone II in Figure 3C with the deduced consensus sequence (**Cons**). Subrepeats A-I and A-2 are marked by red rectangles, and subrepeats B-I, B-2, and B-3 are marked by blue rectangles. Sequence alignment, the consensus sequence (**Cons**), and sequence logos (**Logo**) were produced using CLC Main Workbench (Version 6.9.2, Qiagen).

Supplementary Figure 6 Schematic representation of 5S rDNA organization and the strategy used to generate DNA fragments specific for this genome region in *Pistia stratiotes*. The 5S rDNA repeats were cloned as single, double, or triple units following PCR amplification using primers specific for the 5S rRNA gene sequence (marked as F1, F2 and R1, R2) or to the newly discovered sequences of the NTS (F3 and R3).

Supplementary Figure 7 Alignment of the complete 5S rDNA sequence of *P. stratiotes*. Matching residues are shown as dots. Gap fractions are highlighted in blue.

Supplementary Figure 8 Nucleotide alignment of Type I-a, I-b, I-c, I-d, II-a, and II-b 5S NTS sequences from *P. stratiotes*. Variable residues and regions are highlighted in blue. The accession numbers of the representative sequences deposited in GenBank are as follows: Ps19: OL409055; Ps20: OL409052; Ps22: OL409056; Ps28: OL409060; Ps29: OL409057; Ps30: OL409058; Ps31: OL409059; Ps32: OL409061; Ps33: OL409062; Ps34: OL409063; Ps35: OL409064; Ps36: OL409065.

Supplementary Figure 9 Patterns of G-quadruplex structures predicted for Type II 5S rDNA NTS sequences of *P. stratiotes*. Patterns of G-quadruplex structures predicted for Type II-a **(A)**, represented by clone Ps19 (accession no. OL409055) and Type II-b **(B)**, represented by clone Ps20 (accession no. OL409052). The heights of the peaks indicate the relative strength of each G-quadruplex structure; numbers indicate nucleotide positions relative to the transcription start of the 5S rDNA gene; the filled box corresponds to the position of the sequence TCCTCCCCTCCCTCGACAT in Figure 5C.

Supplementary Figure 10 Sequence alignment of three PCR fragments containing the canonical 5S rDNA gene sequence (**Ps9**) and pseudogenes (**Ps20** and **Ps21)**. The 5S rDNA gene variants are marked by green rectangles. The identifications of the sequences are as follows: Ps9: OL450405; Ps20: OL409052; Ps21: OL409053.

Supplementary Figure 11 Sequence alignment of the six PCR fragments amplified with primers specific for Type I-a and Type I-c NTSs. Type I-a NTS sequences are marked by red rectangles, 5S rDNA by green rectangles, and Type I-c NTSs by blue rectangles. The identifications of the representative sequences are as follows: Ps62—OL409066; Ps63—OL409067.
