# Supplementary material for: The Ribosomal DNA Loci of the Ancient Monocot Pistia stratiotes L. (Araceae) Contain Different Variants of the 35S and 5S Ribosomal RNA Gene Units
Source: Front Plant Sci. 2022 Mar 3;13:819750. doi: 10.3389/fpls.2022.819750 (PMC8928438; doi:10.3389/fpls.2022.819750)
Supplement: Supplementary Figure 1 — General organization of plant 35S rDNA repeats and representation of the cloned fragments of Pistia stratiotes rDNA units characterized in this study. Two genomic DNA regions encoding the major parts of 18S-5.8S-25S rRNA genes (Pi-rDNA-1 and Pi-rDNA-2) and a DNA region containing the 3′-part of the 25S rDNA, intergenic spacer (IGS), and 5′-part of the 18S rDNA (Pi-IGS-1) were cloned as XbaI + MfeI restriction fragments. Ten additional fragments covering the IGS region (Pi-IGS_1 to Pi-IGS-10) were produced by PCR using primers specific for the coding sequences of 25S rRNA (F1) and 18S rRNA (R1) and through-sequenced with internal primers F2, F3, R2, and R3. [file Presentation_1.PPT]

## Slide 1
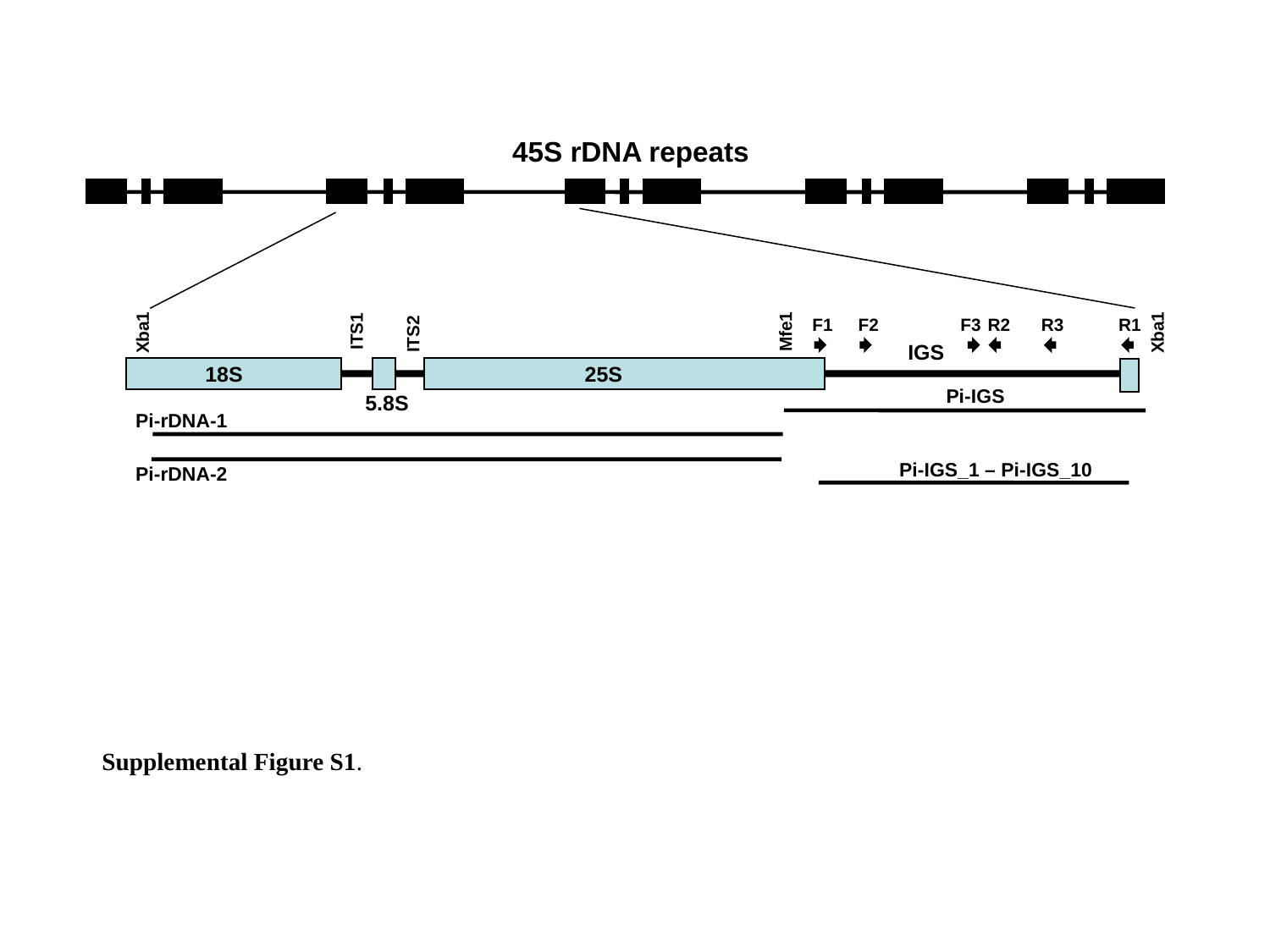

45S rDNA repeats
ITS1
Mfe1
Xba1
Xba1
IGS
18S
 25S
F1
F2
F3
R2
R3
R1
ITS2
Pi-IGS
 5.8S
Pi-rDNA-1
Pi-IGS_1 – Pi-IGS_10
Pi-rDNA-2
Supplemental Figure S1.

## Slide 2
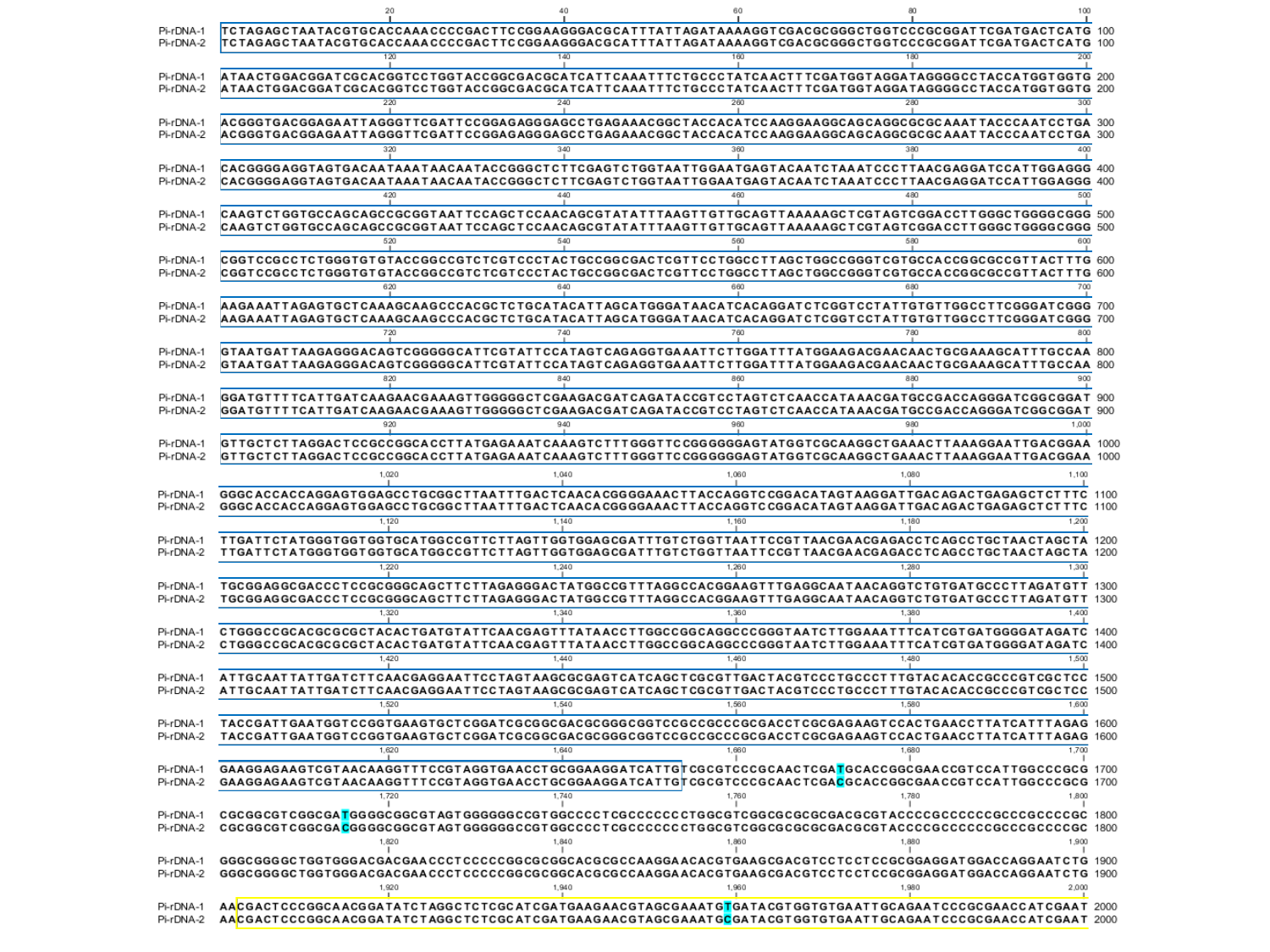

## Slide 3
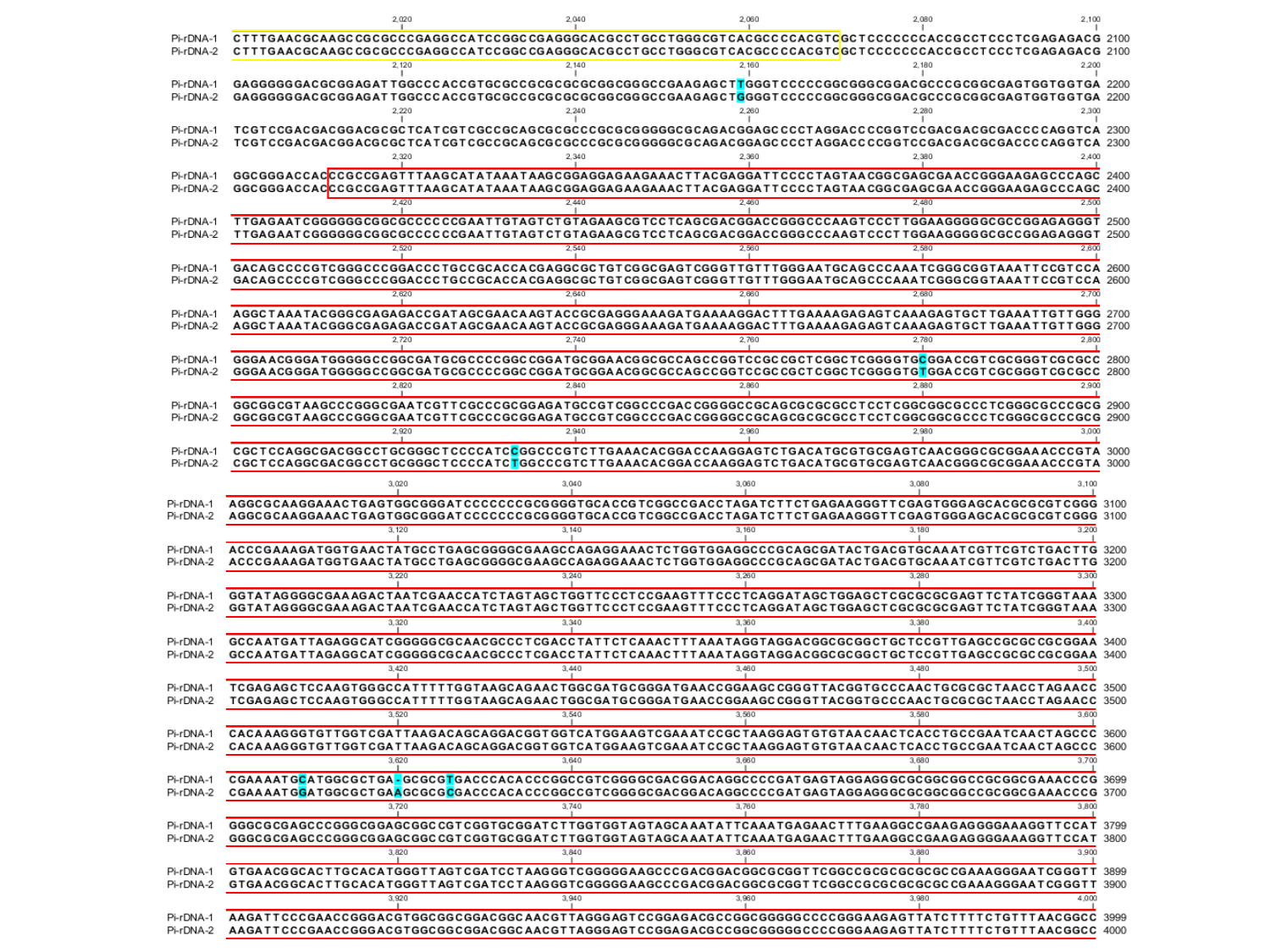

## Slide 4
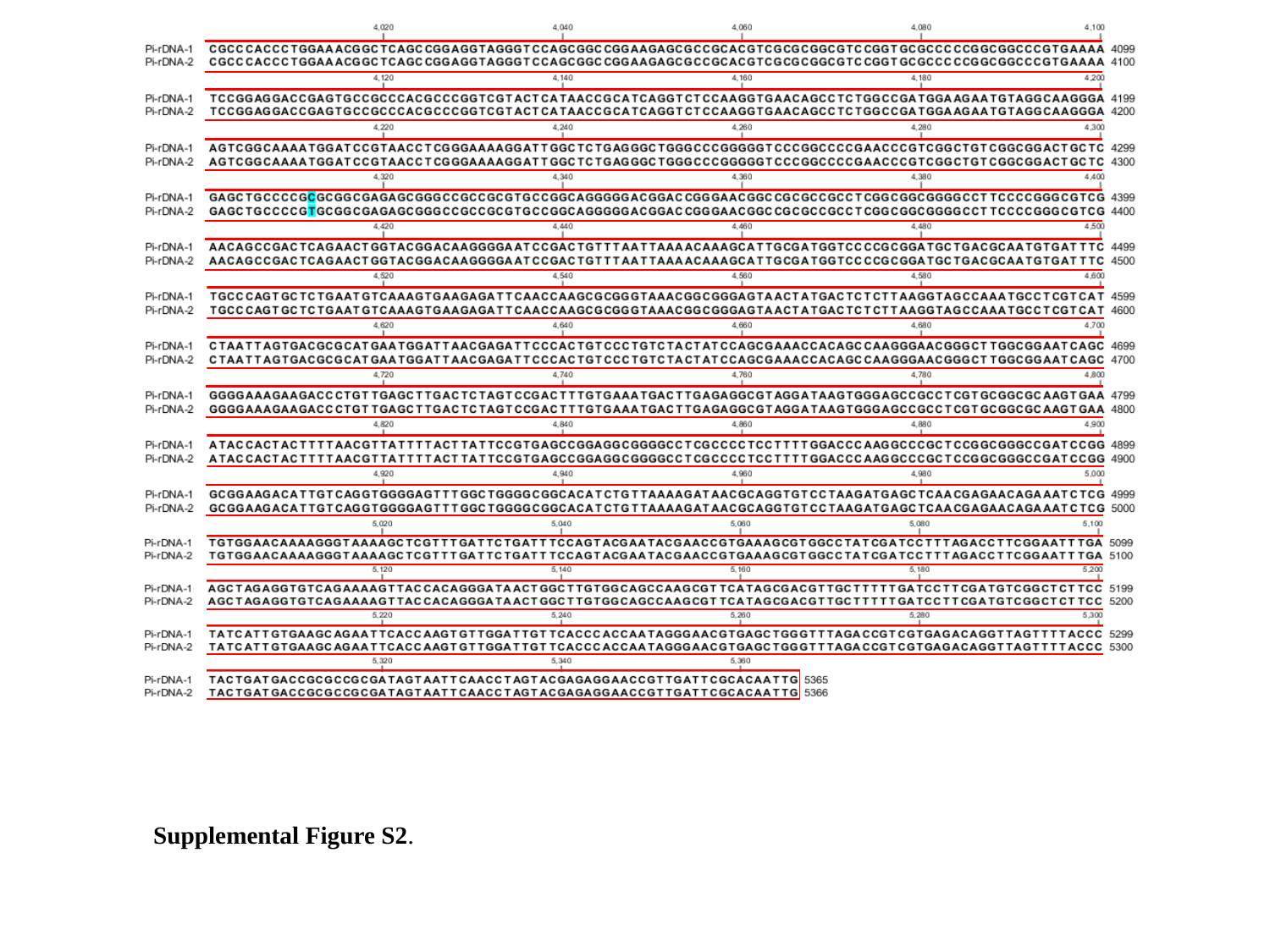

Supplemental Figure S2.

## Slide 5
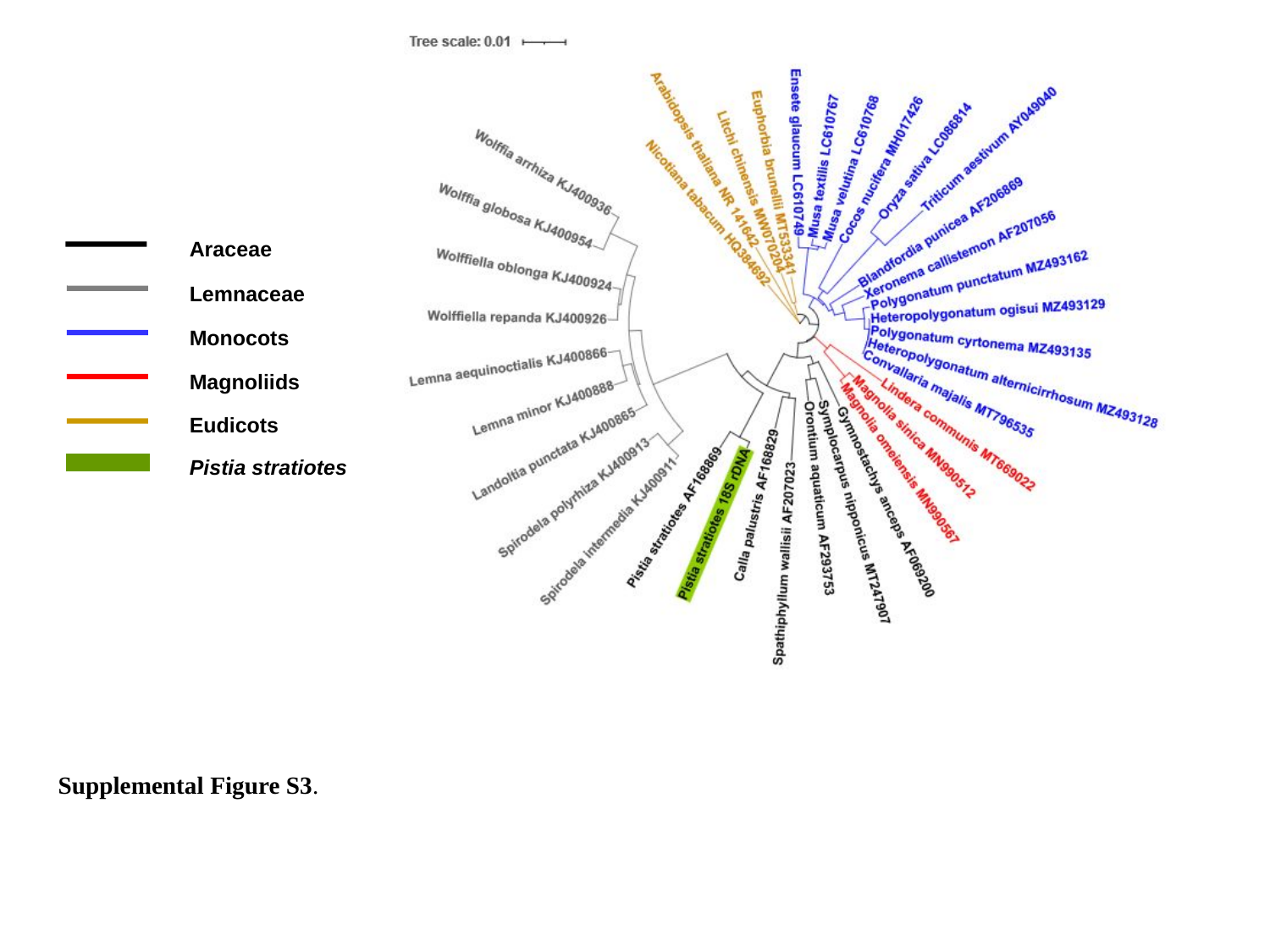

Araceae
Lemnaceae
Monocots
Magnoliids
Eudicots
Pistia stratiotes
Supplemental Figure S3.

## Slide 6
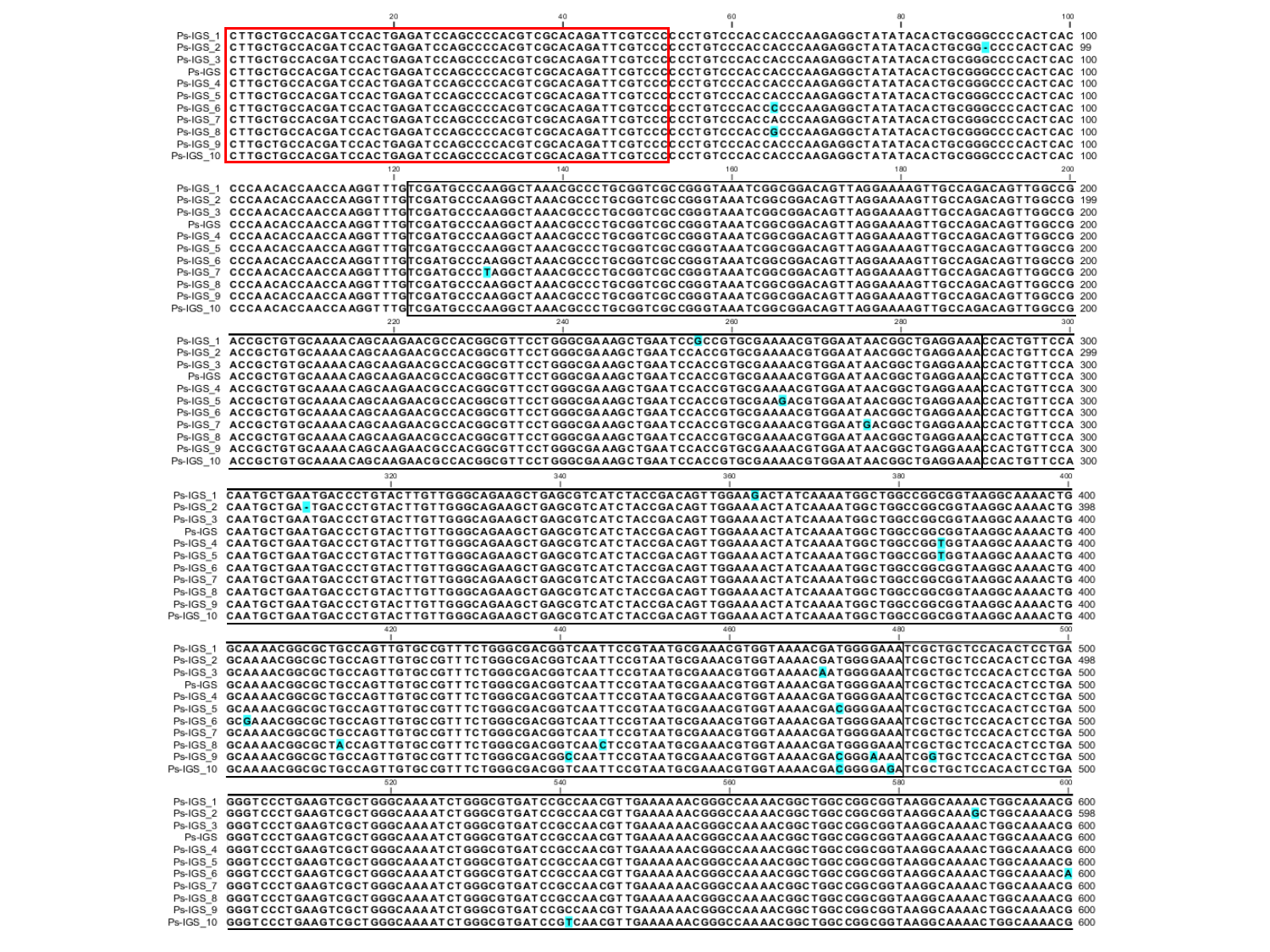

## Slide 7
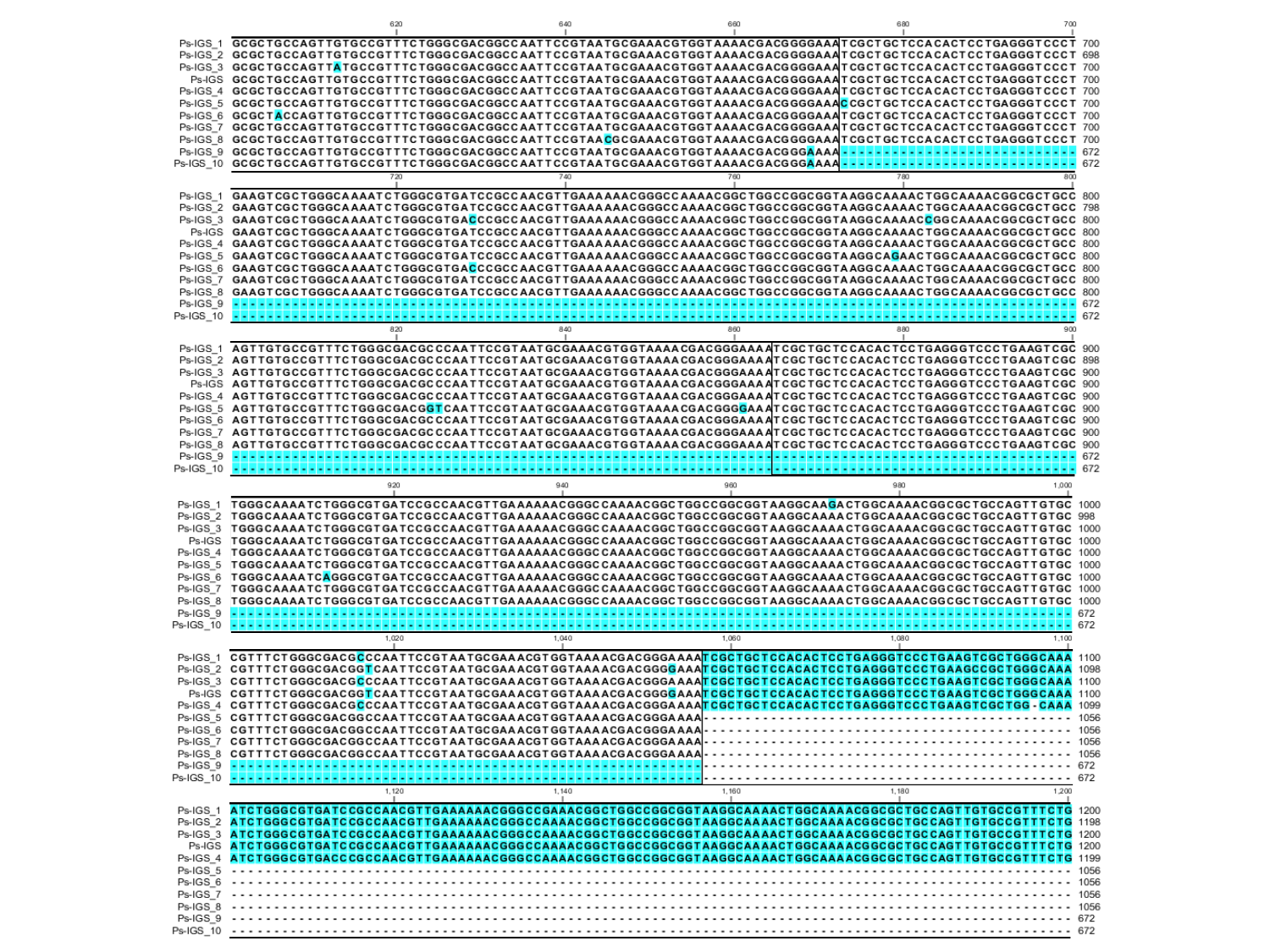

## Slide 8
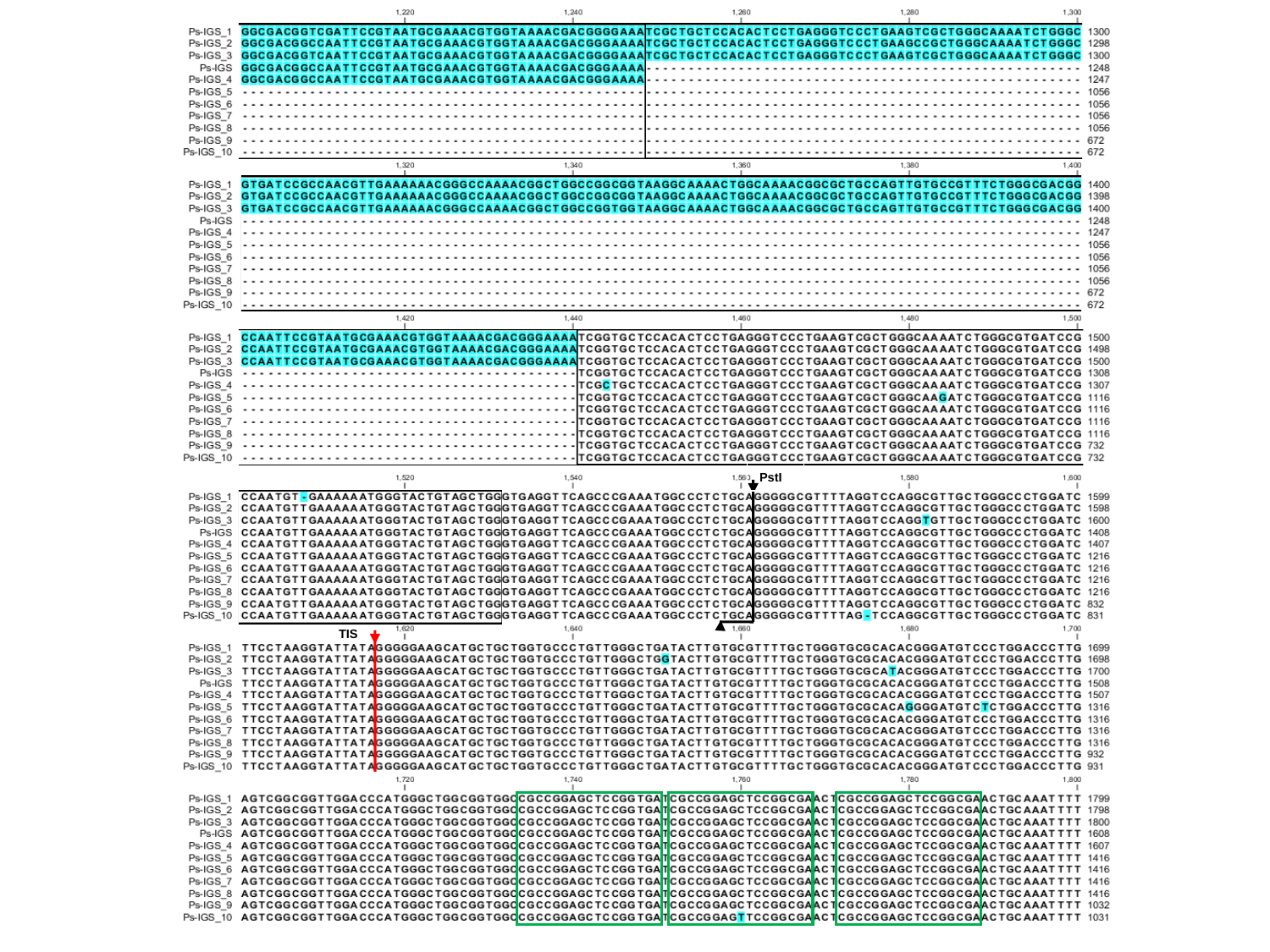

PstI
TIS

## Slide 9
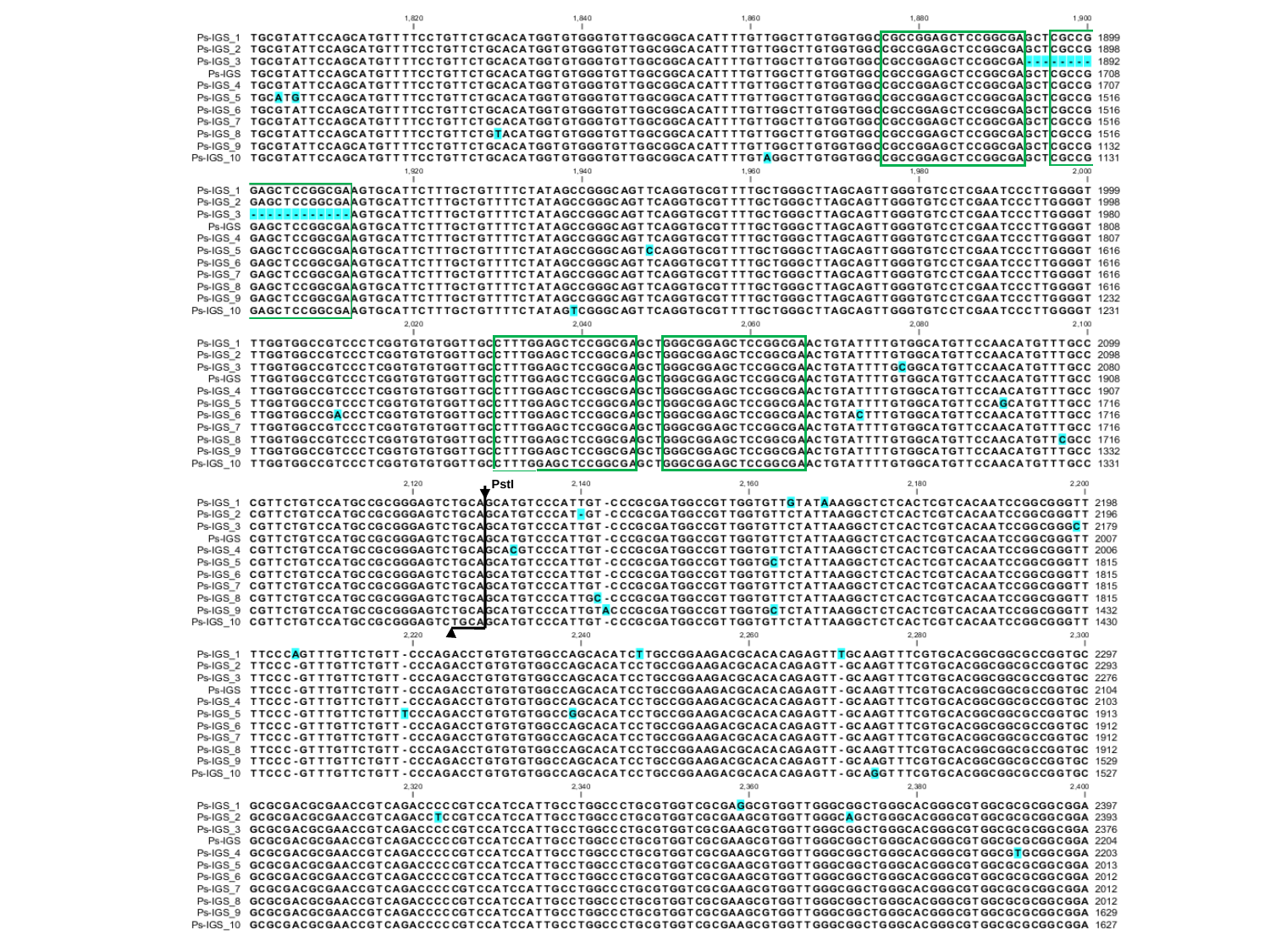

PstI

## Slide 10
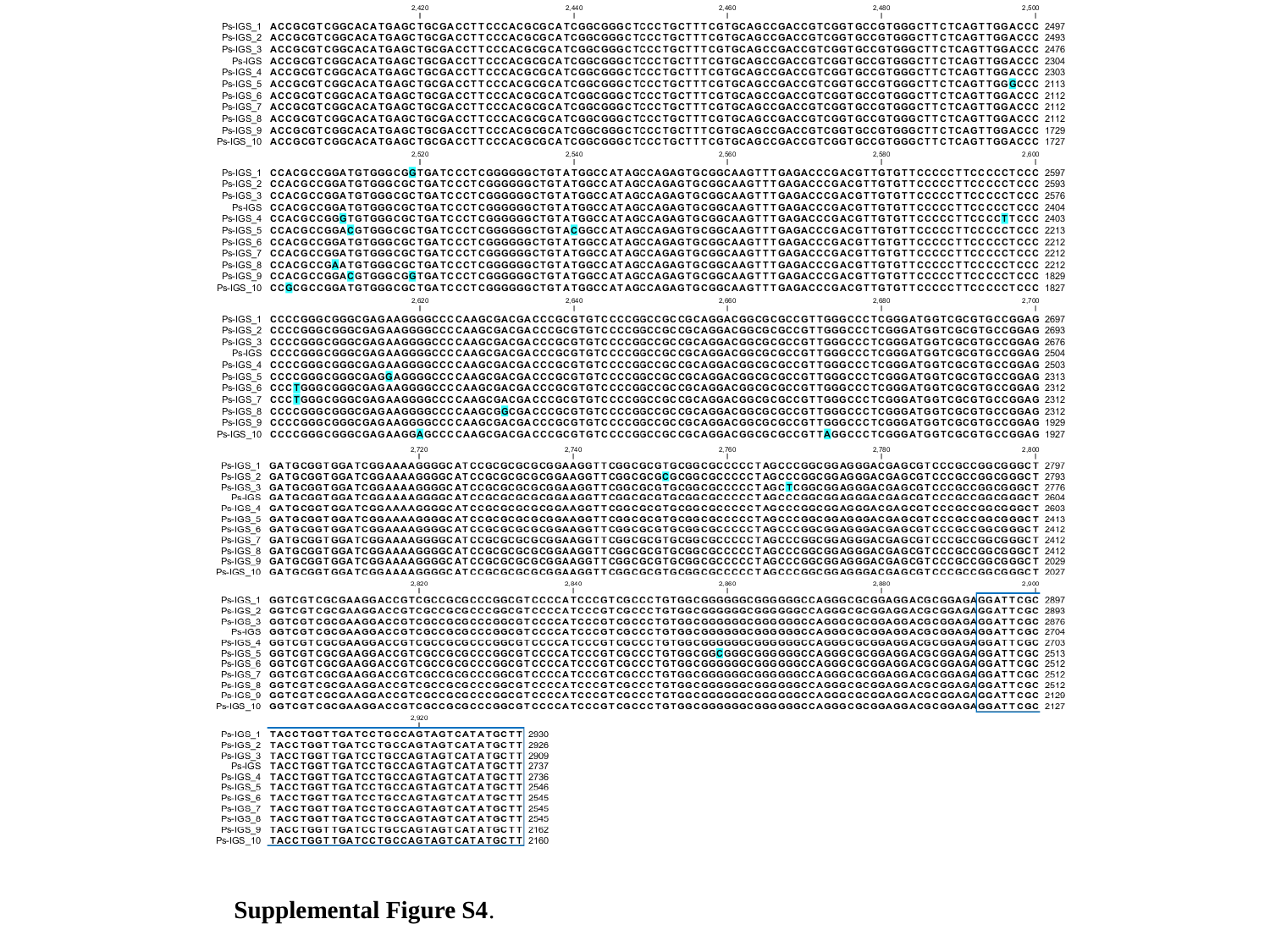

Supplemental Figure S4.

## Slide 11
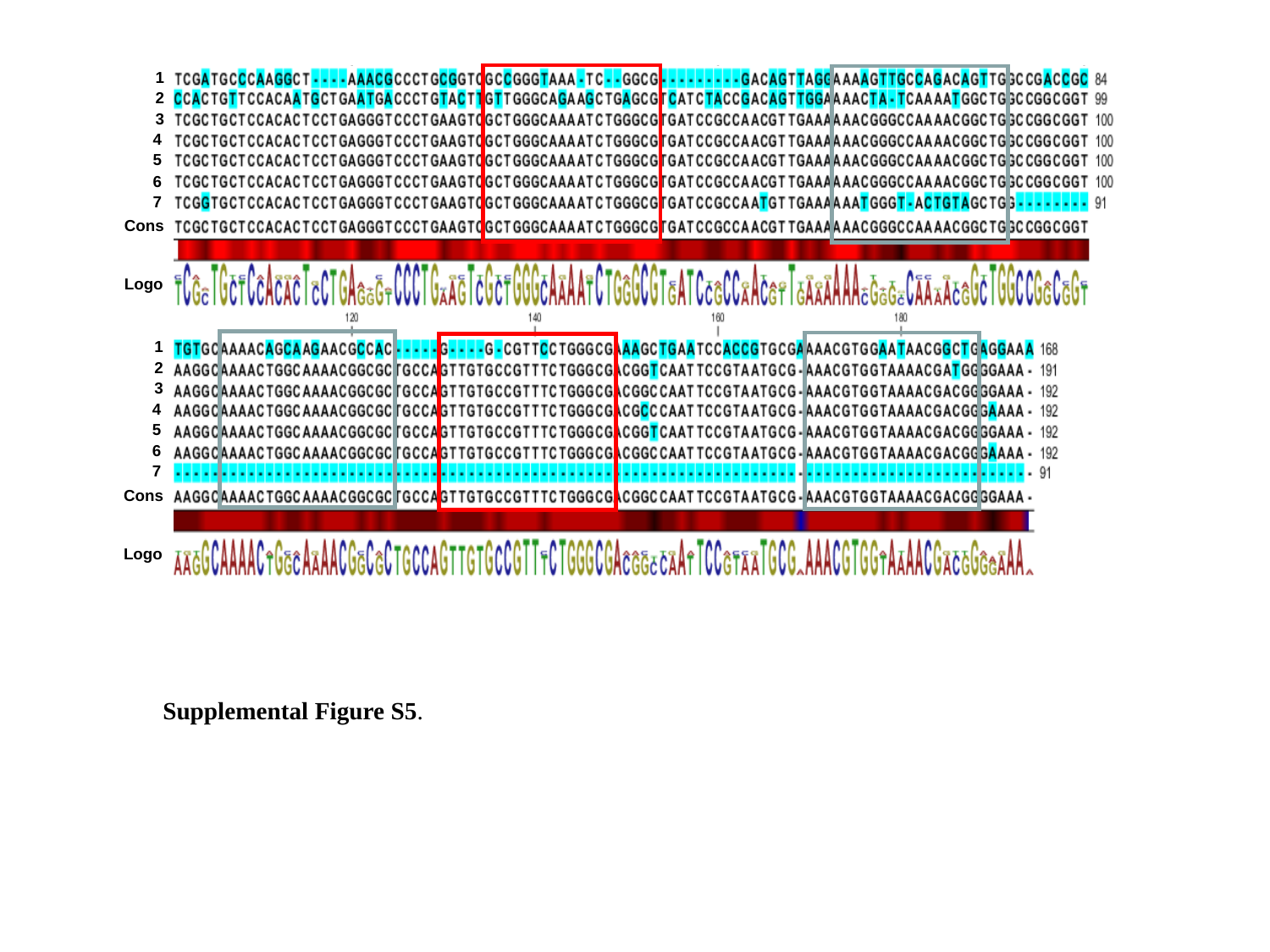

1
2
3
4
5
6
7
Cons
Logo
1
2
3
4
5
6
7
Cons
Logo
Supplemental Figure S5.

## Slide 12
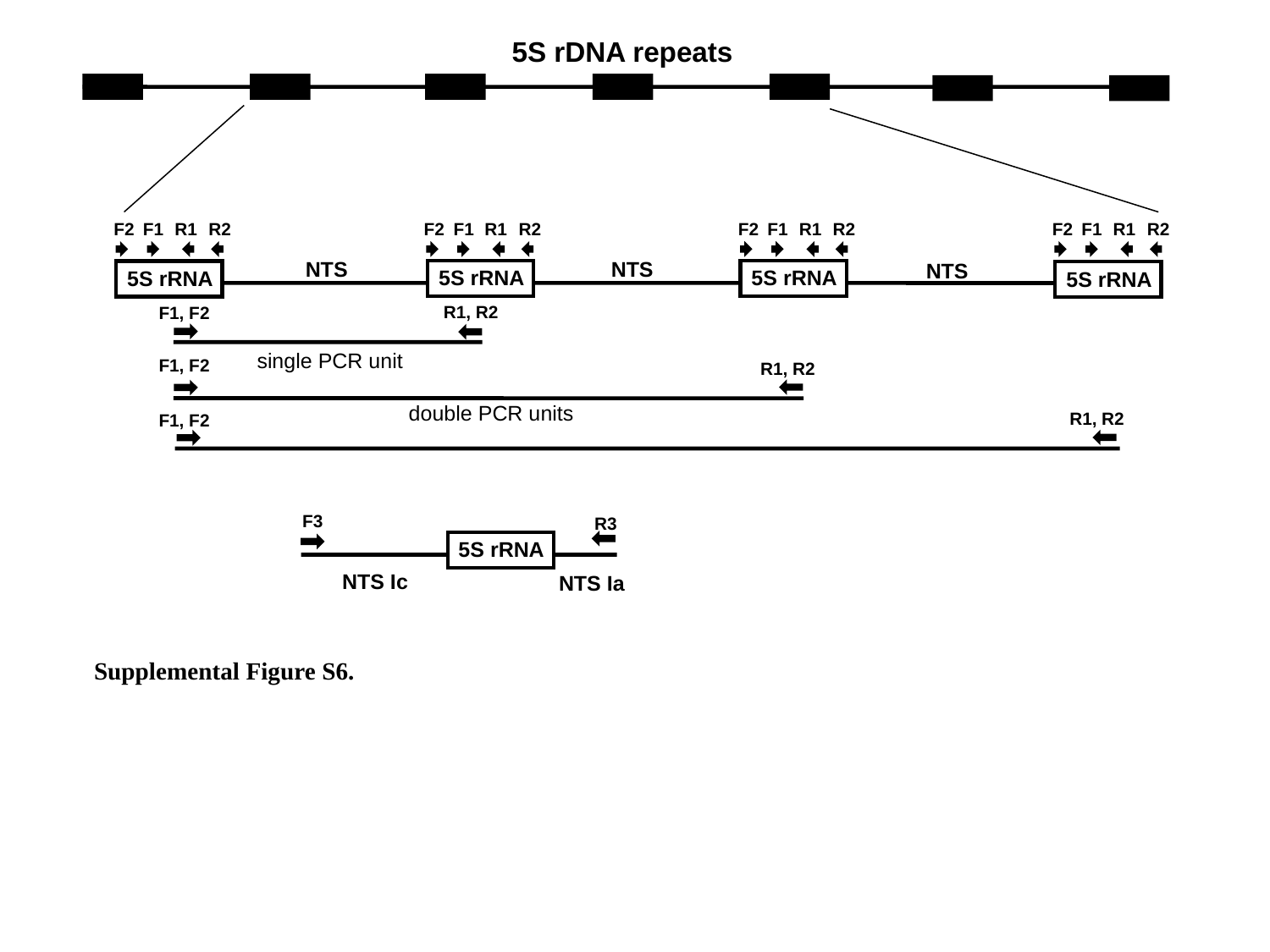

5S rDNA repeats
F2
F1
R1
R2
F2
F1
R1
R2
F2
F1
R1
R2
F2
F1
R1
R2
NTS
NTS
NTS
5S rRNA
5S rRNA5
5S rRNA
5S rRNA5
5S rRNA
5S rRNA5
5S rRNA
5S rRNA5
R1, R2
F1, F2
single PCR unit
F1, F2
R1, R2
double PCR units
R1, R2
F1, F2
F3
R3
5S rRNA
5S rRNA5
NTS Ic
NTS Ia
Supplemental Figure S6.

## Slide 13
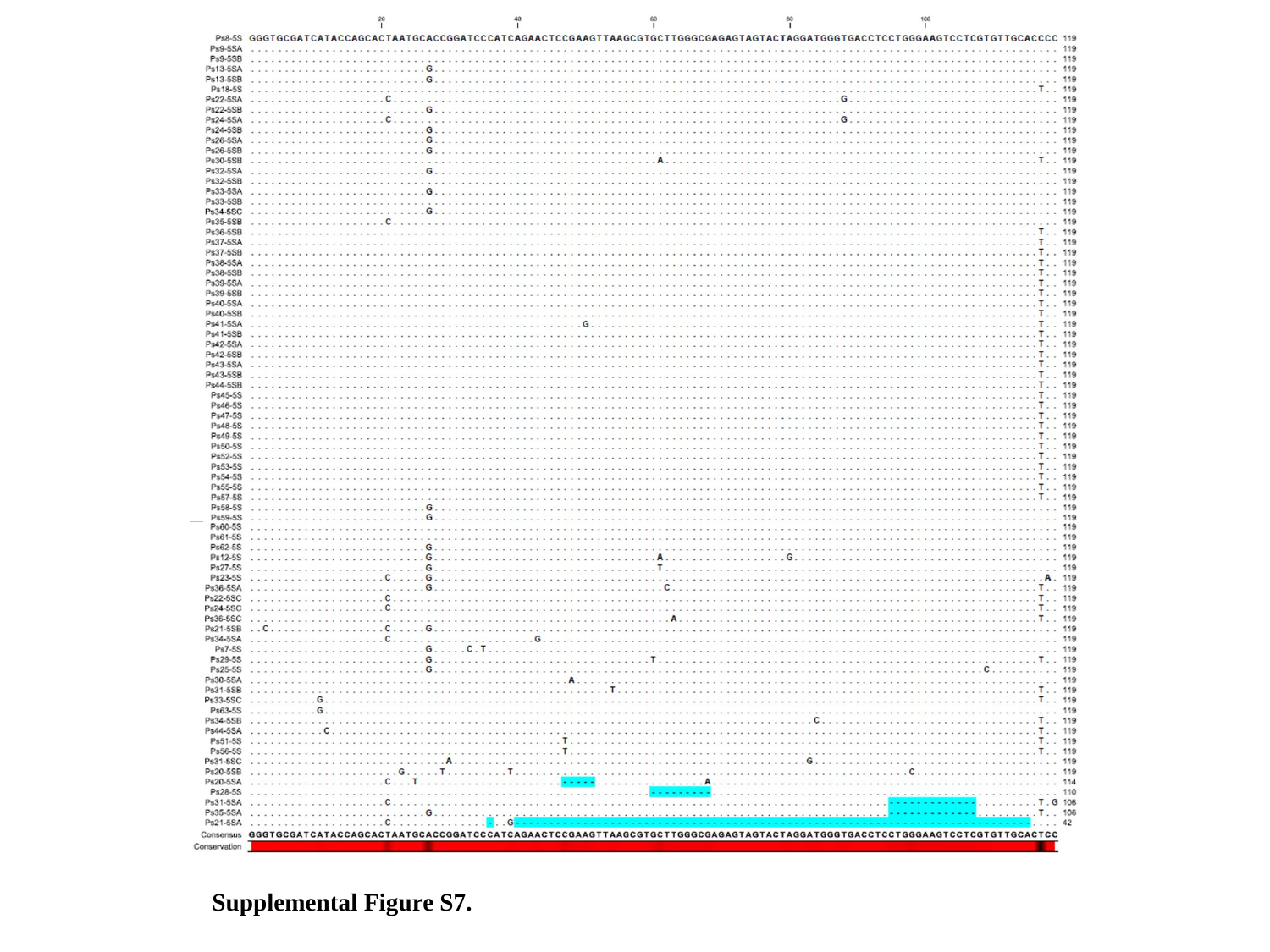

Supplemental Figure S7.

## Slide 14
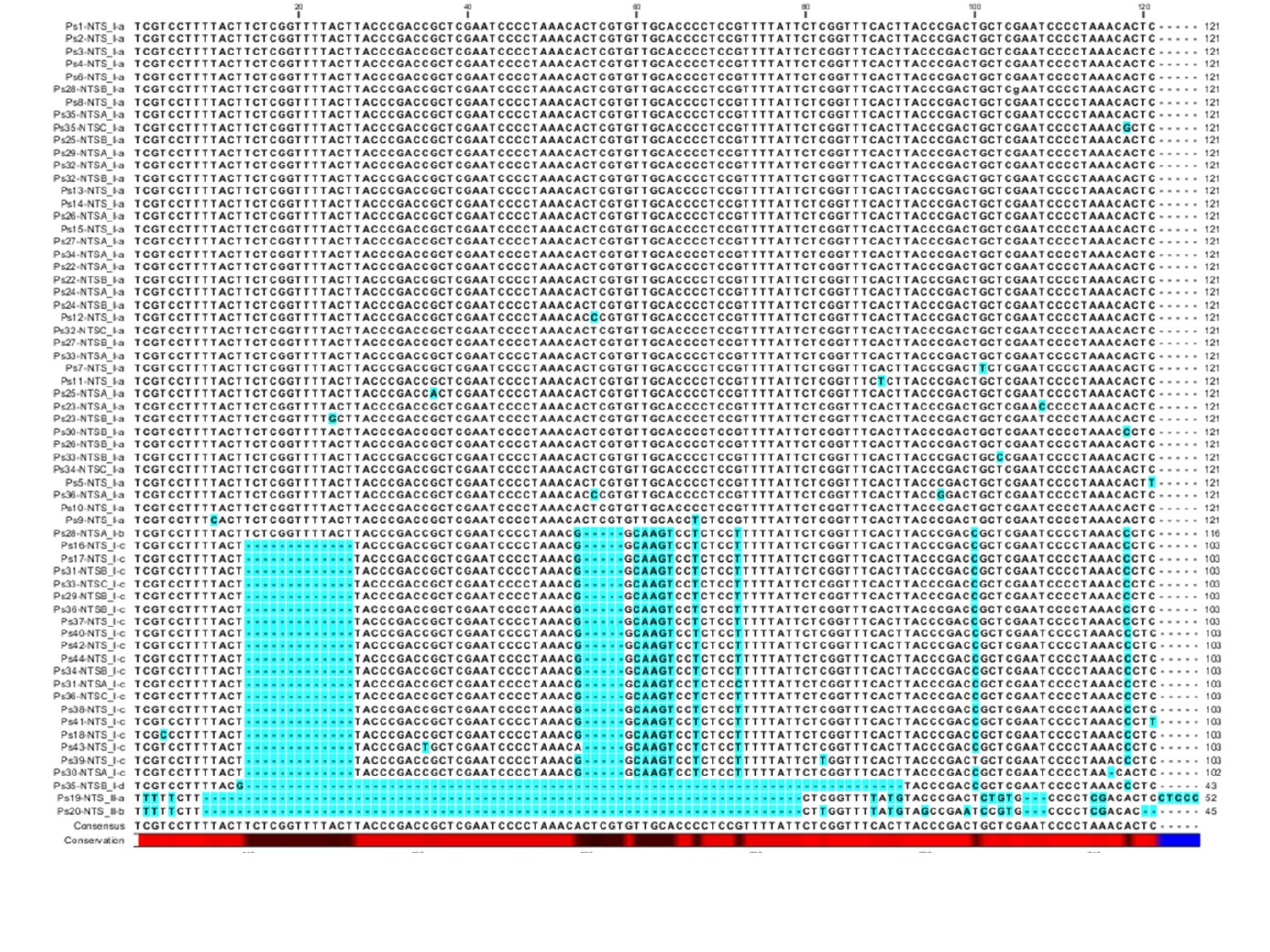

## Slide 15
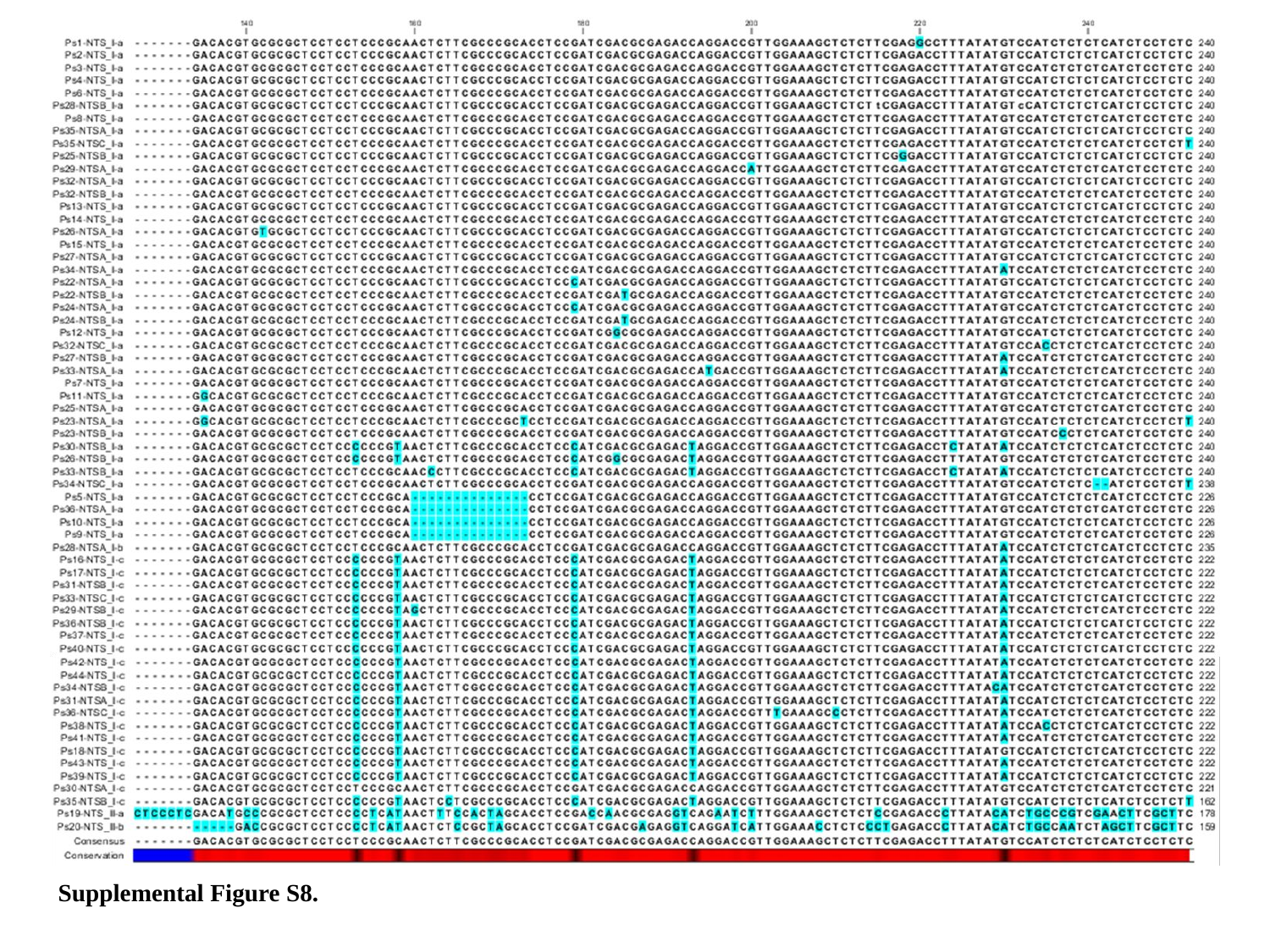

Supplemental Figure S8.

## Slide 16
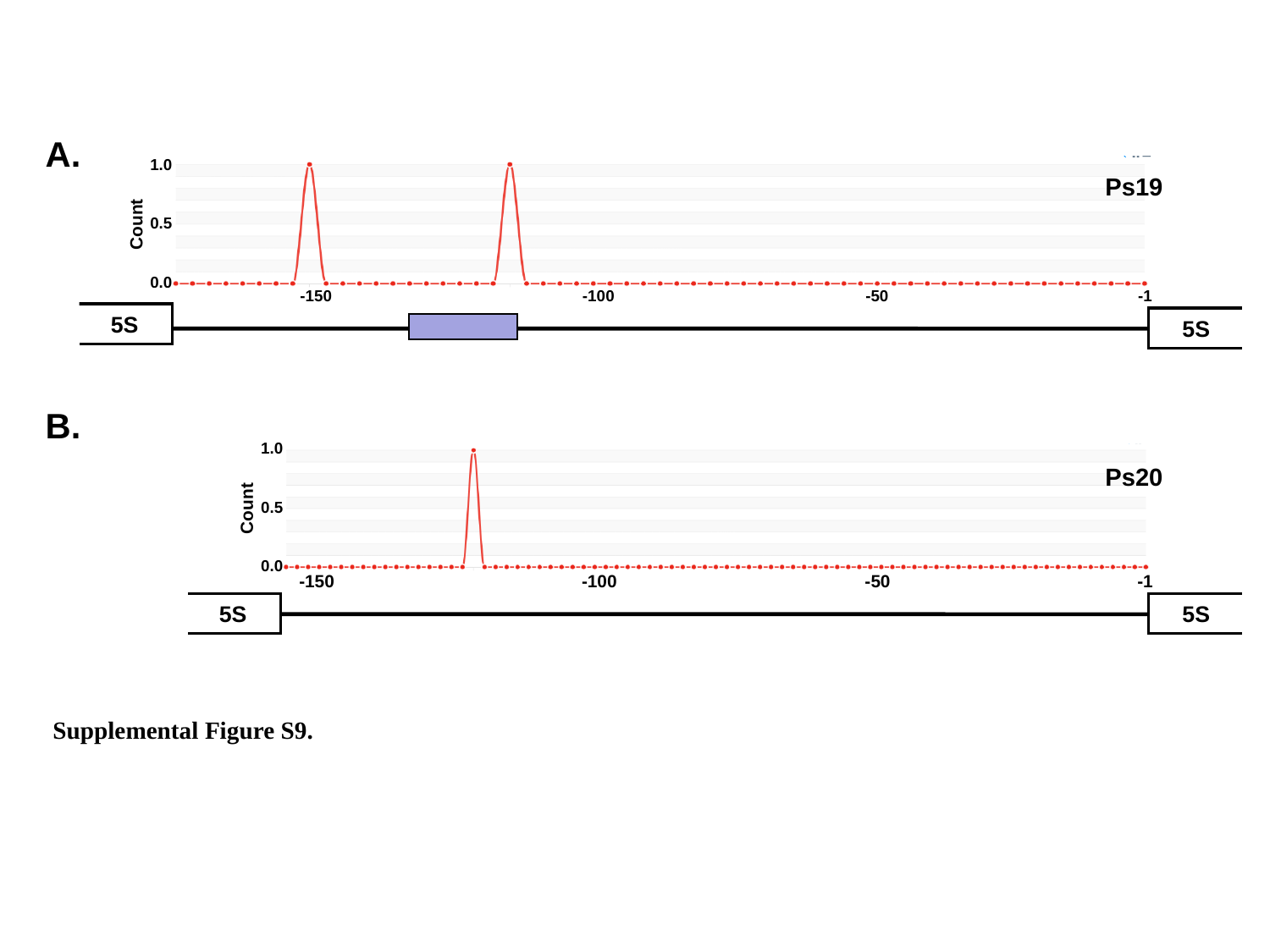

A.
1.0
Count
0.5
0.0
-150
-100
-50
-1
Ps19
5S
5S
B.
1.0
Count
0.5
0.0
-150
-100
-50
-1
Ps20
5S
5S
Supplemental Figure S9.

## Slide 17
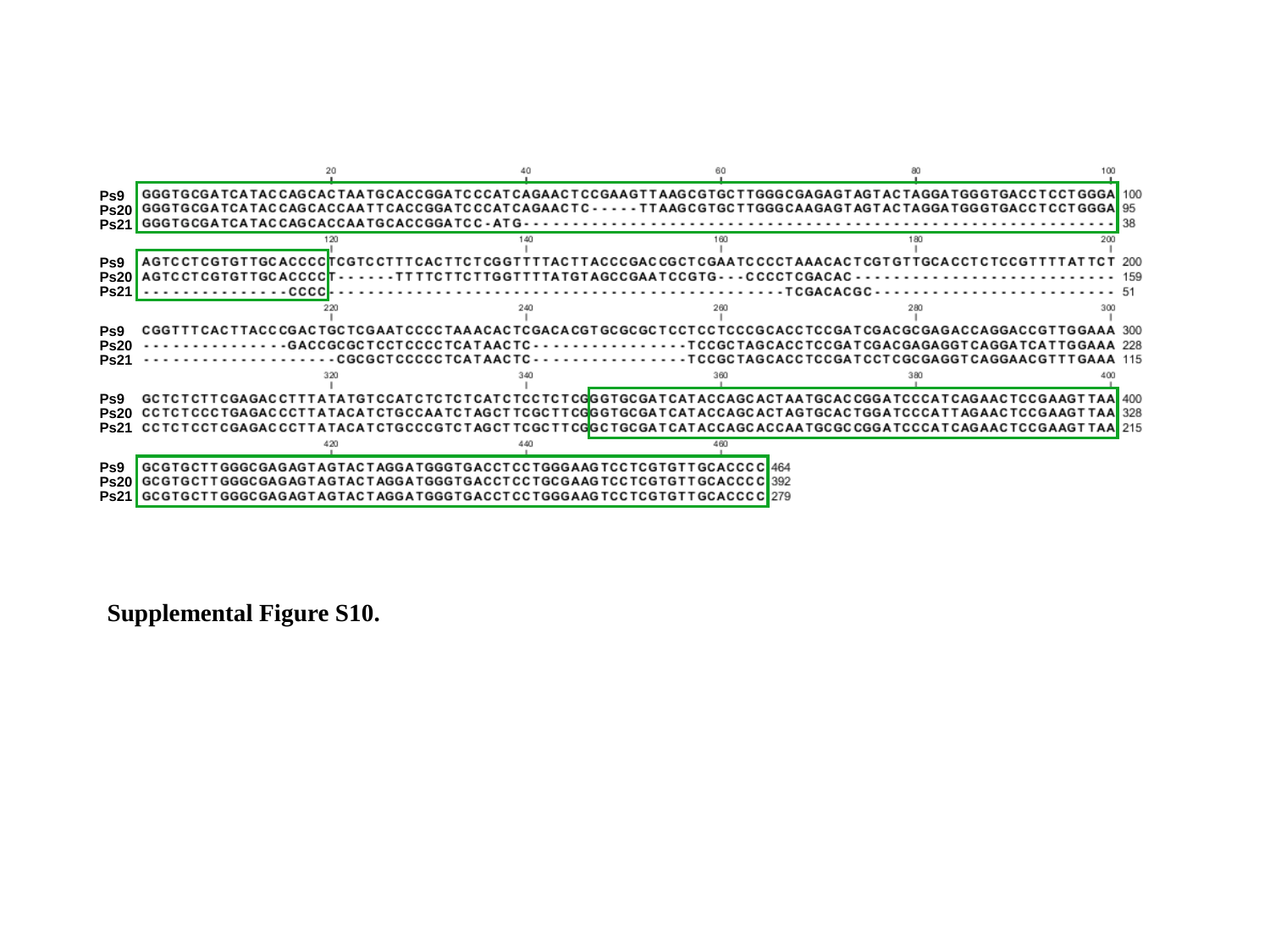

Ps9
Ps20
Ps21
Ps9
Ps20
Ps21
Ps9
Ps20
Ps21
Ps9
Ps20
Ps21
Ps9
Ps20
Ps21
Supplemental Figure S10.

## Slide 18
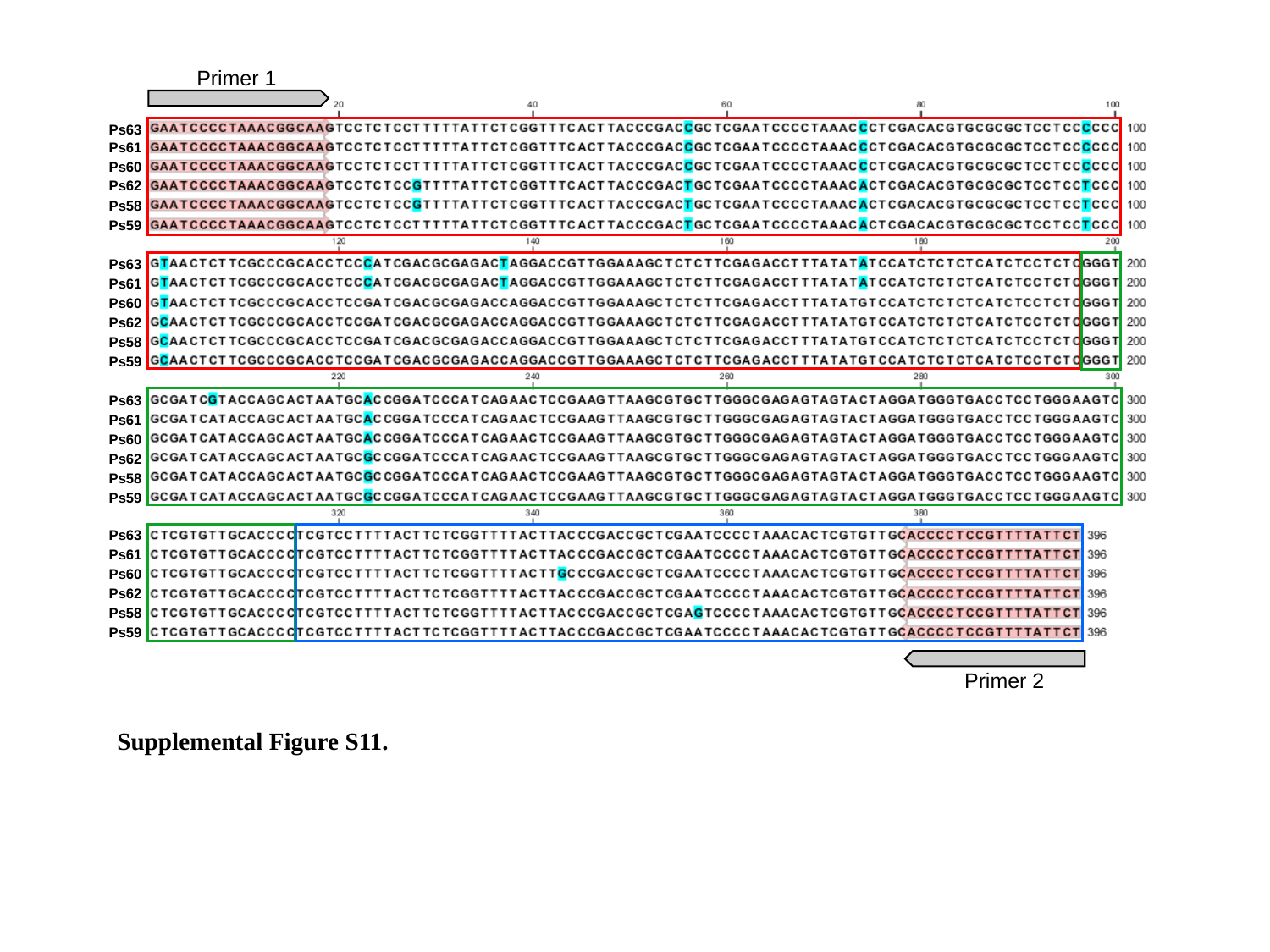

Primer 1
Ps63
Ps61
Ps60
Ps62
Ps58
Ps59
Ps63
Ps61
Ps60
Ps62
Ps58
Ps59
Ps63
Ps61
Ps60
Ps62
Ps58
Ps59
Ps63
Ps61
Ps60
Ps62
Ps58
Ps59
Primer 2
Supplemental Figure S11.
