# Supplementary material for: The Ribosomal DNA Loci of the Ancient Monocot Pistia stratiotes L. (Araceae) Contain Different Variants of the 35S and 5S Ribosomal RNA Gene Units
Source: Front Plant Sci. 2022 Mar 3;13:819750. doi: 10.3389/fpls.2022.819750 (PMC8928438; doi:10.3389/fpls.2022.819750)
Supplement: Supplementary file 2 [file Table_1.DOCX]

**Supplementary Table 1**. List of primers used in this study.

| **Primer ID** | **Sequence, 5’→3’** | **Direction** | **Target** | **Used for** |
| --- | --- | --- | --- | --- |
| **Probe labelling** | | | | |
| Lab-F1 | GTGCGATCATACCAGCACTAATGCACCGG | Forward | 5S rDNA | rDNA probe labelling |
| Lab-R1 | GAGGTGCAACACGAGGACTTCCCAGGAGG | Reverse | 5S rDNA | rDNA probe labelling |
| **45S rDNA** | | | | |
| 25S-F | GTGTAACAACTCACCTGCCG | Forward | 25S rDNA | Screening genomic clones and sequencing IGS |
| 25S-R | AAACAGTCGGATTCCCCTTGTCC | Reverse | 25S rDNA | Screening genomic clones and sequencing 25S rDNA |
| 18S-F1 | GGCGGATGTTGCTCTTAGG | Forward | 18S rDNA | Sequencing 18S rDNA |
| ITS1f-A | CGATTGAATGGTCCGGTGAAG | Forward | 18S rDNA | Sequencing ITS 1 |
| ITS1f-B | ACCTTATCATTTAGAGGAAG**G** | Forward | 18S rDNA | Sequencing ITS 1 |
| 5.8S-F1 | CATCGATGAAGAACGCAGCG | Forward | 5.8S rDNA | Sequencing ITS 2 |
| 5.8S-R1 | GCGTTCAAAGATTCGATGG | Reverse | 5.8S rDNA | Sequencing ITS 1 |
| ITS2r-A | TCGCCGTTACTAGGGGAATC | Reverse | 25S rDNA | Sequencing ITS 2 |
| ITS2r-B | TCTACAGACTACAATTCGG | Reverse | 25S rDNA | Sequencing ITS 2 |
| 25S-F1 | GAGAGTCAAAGAGTGCTTG | Forward | 25S rDNA | Sequencing 25S rDNA |
| 25S-R1 | AAACAGTCGGATTCCCCTTGTCC | Reverse | 25S rDNA | Sequencing ITS 2 |
| Clo25Sfor | CAATTGCCGTCCCGCAGTAGG | Forward | 25S rDNA | Screening genomic clones containing 45S rDNA IGS |
| Clo25Srev | GAGGGACGAATCTGTGCGACG | Reverse | 25S rDNA | Screening genomic clones containing 45S rDNA IGS |
| Clo18Sfor | GGACGTGCTACCTGGTTGATCC | Forward | 18S rDNA | Screening genomic clones containing 45S rDNA IGS |
| Clo18Srev | CTACGGTTATCCGAGTAGCAGG | Reverse | 18S rDNA | Screening genomic clones containing 45S rDNA IGS |
| 25S-f3 | CTTGCTGCCACGATCCACTGAG | Forward | 25S rDNA | Sequencing 45S rDNA NTS |
| Pi-IGS-F2 | TGGAAAACTATCAAAATGGC | Forward | 45S rDNA NTS | Internal primer for sequencing 45S rDNA NTS |
| Pi-IGS-F3 | TTTTAGGTCCAGGCGTTGC | Reverse | 45S rDNA NTS | Internal primer for sequencing 45S rDNA NTS |
| Pi-IGS-R1 | GGAAAACCCGCCGGATTGTGAC | Forward | 45S rDNA NTS | Internal primer for sequencing 45S rDNA NTS |
| Pi-IGS-R2 | AATACCTTAGGAAGATCC | Reverse | 45S rDNA NTS | Internal primer for sequencing 45S rDNA NTS |
| 18S-R | CCATTCGCAGTTTCACAGTCC | Reverse | 18S rDNA | Sequencing 45S rDNA NTS |
| **5S rDNA** | | | | |
| DW-5S-F | CTTGGGCGAGAGTAGTACTAGG | Forward | 5S rDNA | 5S rDNA repeats cloning |
| DW-5S-R | CACGCTTAACTTCGGAGTTCTG | Reverse | 5S rDNA | 5S rDNA repeats cloning |
| Cn-5S-F | GGGTGCGATCATACCAGCAC | Forward | 5S rDNA | 5S rDNA repeats cloning |
| Cn-5S-R | GGGGTGCAACACGAGGACTTC | Reverse | 5S rDNA | 5S rDNA repeats cloning |
| Ps5SIGSIIF1 | GAATCCCCTAAACGGCAAG | Forward | 5S rDNA NTS | 5S rDNA repeats cloning |
| Ps5SIGSIR1 | AGAATAAAACGGAGGGGTG | Reverse | 5S rDNA NTS | 5S rDNA repeats cloning |
